# Enhancement of Atmospheric Nucleation Precursors on Iodic Acid-Induced Nucleation: Predictive Model and Mechanism

**DOI:** 10.1021/acs.est.3c01034

**Published:** 2023-04-21

**Authors:** Fangfang Ma, Hong-Bin Xie, Rongjie Zhang, Lihao Su, Qi Jiang, Weihao Tang, Jingwen Chen, Morten Engsvang, Jonas Elm, Xu-Cheng He

**Affiliations:** † Key Laboratory of Industrial Ecology and Environmental Engineering (Ministry of Education), School of Environmental Science and Technology, Dalian University of Technology, Dalian 116024, China; ‡ National-Regional Joint Engineering Research Center for Soil Pollution Control and Remediation in South China, Guangdong Key Laboratory of Integrated Agro-environmental Pollution Control and Management, Institute of Eco-environmental and Soil Sciences, Guangdong Academy of Sciences, Guangzhou 510650, China; § Department of Chemistry and iClimate, Aarhus University, Langelandsgade 140, DK-8000 Aarhus C, Denmark; ∥ Institute for Atmospheric and Earth System Research/Physics, University of Helsinki, Helsinki 00014, Finland; ⊥ Finnish Meteorological Institute, Helsinki 00560, Finland

**Keywords:** marine particle formation, iodic acid, QSAR, diethylamine, quantum chemical calculation, atmospheric cluster dynamics simulation

## Abstract

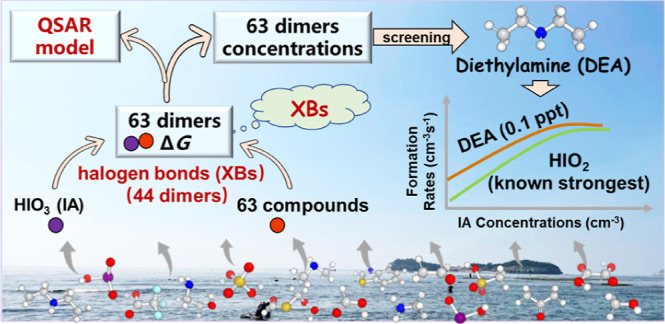

Iodic acid (IA) has recently been recognized as a key driver for new particle formation (NPF) in marine atmospheres. However, the knowledge of which atmospheric vapors can enhance IA-induced NPF remains limited. The unique halogen bond (XB)-forming capacity of IA makes it difficult to evaluate the enhancing potential (EP) of target compounds on IA-induced NPF based on widely studied sulfuric acid systems. Herein, we employed a three-step procedure to evaluate the EP of potential atmospheric nucleation precursors on IA-induced NPF. First, we evaluated the EP of 63 precursors by simulating the formation free energies (Δ*G*) of the IA-containing dimer clusters. Among all dimer clusters, 44 contained XBs, demonstrating that XBs are frequently formed. Based on the calculated Δ*G* values, a quantitative structure–activity relationship model was developed for evaluating the EP of other precursors. Second, amines and O/S-atom-containing acids were found to have high EP, with diethylamine (DEA) yielding the highest potential to enhance IA-induced nucleation by combining both the calculated Δ*G* and atmospheric concentration of considered 63 precursors. Finally, by studying larger (IA)_1–3_(DEA)_1–3_ clusters, we found that the IA-DEA system with merely 0.1 ppt (2.5×10^6^ cm^–3^) DEA yields comparable nucleation rates to that of the IA–iodous acid system.

## Abstract 2

Based on the calculated dimer formation free energies of 63 precursors with iodic acid (IA), a predictive model of enhancing potential was developed, and the strongest enhancer for IA-induced nucleation was identified to be diethylamine.

## Introduction

Marine aerosols play a significant role in the climate system by directly scattering solar radiation and indirectly forming clouds. Both primary aerosols, such as sea spray aerosols, and secondary aerosols formed by new particle formation (NPF) contribute to the formation of cloud condensation nuclei.^1,2^ The relative contribution of these two channels has been under debate, but recent studies have pointed out the larger contribution from particle formation processes.^3,4^ Unfortunately, the understanding of NPF mechanisms over the marine atmosphere remains limited. Sulfuric acid (SA) and methane sulfonic acid (MSA) are commonly believed to be the driving forces for marine NPF, and they have both been extensively studied.^5−13^ In recent years, field observations have identified iodic acid (HIO_3_, IA) as another prominent candidate for marine aerosol nucleation in distinct environments.^14−17^ However, compared to SA- and MSA-induced nucleation mechanisms, the IA-induced nucleation mechanism is poorly understood. Considering the important role of IA in polar and marine environments, it is necessary to investigate the IA-induced nucleation mechanisms.

As an oxoacid, IA could cluster with atmospheric bases [e.g., ammonia (NH_3_), amines (e.g., monomethylamine MA, dimethylamine DMA, and trimethylamine TMA)] via acid–base reactions, and with other oxoacids via hydrogen bonds (HBs), similar to SA and MSA. In addition, IA could also form halogen bonds (XBs) with O/S/N-atom-containing compounds. Therefore, potential compounds such as amines (e.g., alkyl amines, aromatic amines, amides, and amino acids), organic acids, inorganic oxoacids, etc. could interact with IA and enhance the IA-induced aerosol nucleation in the marine atmosphere. However, the enhancement from only a limited subset of these compounds, i.e., iodous acid (HIO_2_), NH_3_, DMA, SA, and MSA, has been investigated using quantum chemical calculations.^18−22^ Among the investigated compounds, HIO_2_ was found to have the highest enhancement to pure IA nucleation.^18^ However, the current knowledge may not well predict IA-induced nucleation under all circumstances, especially in chemically complex polluted and semi-polluted environments. As the presence of iodine species in polluted environments has been unambiguously revealed,^14,23^ it is necessary to investigate the enhancing potential of other compounds on IA-induced nucleation, with special attention to compounds with stronger enhancing potential compared to HIO_2_.

The formation of IA-containing dimer clusters [(IA)_1_(X)_1_, X representing various precursors] is the key step in IA-induced nucleation, similar to the corresponding SA and MSA-induced nucleation mechanisms.^1,24−26^ Thus, the formation free energy (Δ*G*) of the (IA)_1_(X)_1_ dimer clusters is a crucial parameter to evaluate the nucleation potential of various compounds. In this study, a three-step scheme was adopted to advance the current understanding on IA-induced nucleation. First, quantum chemical methods were used to calculate the Δ*G* values of the (IA)_1_(X)_1_ dimer clusters for 63 selected compounds. All the selected 63 compounds, including iodine and sulfur/nitrogen oxoacids, NH_3_, amines, carbonyl compounds, monocarboxylic acids, dicarboxylic acids, sulfides, thiols, alcohols, sulfones, benzothiazole, peroxides, and hydroperoxymethyl thioformate (see their structures in Figure S1), were detected in the marine atmosphere. Based on the calculated Δ*G* values, a quantitative structure–activity relationship (QSAR) model was constructed. Second, we evaluated the atmospheric concentrations of 63 (IA)_1_(X)_1_ dimer clusters. Finally, based on the concentrations of the (IA)_1_(X)_1_ dimer clusters, the compounds with the highest dimer concentration [diethylamine (DEA)] were selected as the representative to investigate their exact enhancing potential on IA-induced nucleation by considering larger (IA)_*x*_(DEA)_*y*_ (0 ≤ *x* ≤ 3, 0 ≤ *y* ≤ 3) clusters.

## Methodology

### Configurational Sampling

In this study, a multistep global minimum sampling scheme was employed to search for the global minima of the (IA)_1_(X)_1_ dimer clusters and the (IA)_*x*_(DEA)_*y*_ (*x* = 1–3, *y* = 1–3) clusters. This approach has been successfully employed in past theoretical studies on aerosol nucleation.^18,25−33^ As mentioned in our previous study,^18^ the initial conformers of each cluster with *n* molecules were constructed by randomly placing a monomer around cluster minima with *n* – 1 molecules, which were performed in an in-house code. Herein, 1000–4000 and 3000–9000 randomly generated initial structures of (IA)_1_(X)_1_ dimer clusters and (IA)_*x*_(DEA)_*y*_ (*x* = 1–3, *y* = 1–3) clusters, respectively, were optimized at the PM7 level of theory. For all the converged geometries, a single-point energy calculation was performed at the M06-2X/def2-TZVP level of theory. The conformers within 8–10 kcal mol^–1^ compared to the identified lowest energy conformer were further optimized at the M06-2X/6-31++G(d,p) + aug-cc-pVTZ(-PP) level of theory (6-31++G(d,p) for H, N, S, O, and F atoms and aug-cc-pVTZ-PP with ECP28 for the I atom). During the geometry optimization process, if the optimized conformers fail or have imaginary frequencies, the initial geometries will be adjusted and reoptimized until an “acceptable” conformer is obtained. The identified lowest free energy configurations within 1–2 kcal mol^–1^ were reoptimized at the M06-2X/aug-cc-pVTZ(-PP) (aug-cc-pVTZ for H, N, S, O, and F atoms and aug-cc-pVTZ-PP with ECP28 for the I atom) level of theory, followed by the single point energy calculations at the DLPNO-CCSD(T)/aug-cc-pVTZ(-PP) level of theory. Finally, the conformer with the lowest Gibbs free energy (*G*) at 298.15 K was selected as the global minimum for a given cluster. All the PM7 and M06-2X calculations were performed within the GAUSSIAN 16 program package,^34^ and the DLPNO-CCSD(T) calculations were performed using the ORCA 4.0.0 program.^35^ In all DLPNO-CCSD(T) calculations, the keywords “TightPNO, TightSCF, and GRID4” were used. The structures of (IA)_2_, (IA)_1_(HIO_2_)_1_, (IA)_1_(NH_3_)_1_, (IA)_1_(DMA)_1_, (IA)_1_(SA)_1_, (IA)_1_(MSA)_1_, (IA)_1–3_, and (DEA)_1–3_ were obtained from previous studies^18−21,36−38^ and were recalculated at the theoretical level employed here.

The *G* values of the identified clusters at 298.15 K are the sum of electronic single-point energy at the DLPNO-CCSD(T)/aug-cc-pVTZ(-PP) level of theory and the Gibbs free energy correction terms (*G*_corr_) at the M06-2X/aug-cc-pVTZ(-PP) level of theory. In addition, the *G* values at other temperatures were obtained by combining the single-point energies at the DLPNO-CCSD(T)/aug-cc-pVTZ(-PP) level of theory and the recalculated *G*_corr_ values of clusters at the M06-2X/aug-cc-pVTZ(-PP) level of theory using the GoodVibes Python script^39^ at the corresponding temperature. The binding free energy (Δ*G*) for individual clusters is taken as the *G* value of a cluster relative to its constituent monomers at the considered temperature. For the 63 selected compounds with flexible monomer structures, Born–Oppenheimer molecular dynamics simulations based on the density functional theory within the CP2K program package^40^ combined with quantum chemical calculations were used to search for their global minimum structures, and the computational details can be found in our previous study.^41^

### Estimating the Concentrations of (IA)_1_(X)_1_ Dimer Clusters

The atmospheric (IA)_1_(X)_1_ dimer cluster concentrations can offer a reference for evaluating the enhancing potential of X on IA-induced nucleation in the atmosphere. Therefore, we calculated the concentrations of 63 (IA)_1_(X)_1_ dimer clusters [(IA)_1_(X)_1_] based on the Δ*G* values and the concentrations of IA and X to identify the compounds with high potential for enhancing IA-induced nucleation. At equilibrium, the dimer cluster formation rate is equal to the dimer cluster loss rate (evaporation and coagulation sink), hence the [(IA)_1_(X)_1_] can be written as^42,43^
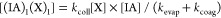
1
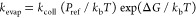
2
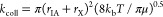
3where *k*_coll_ is the kinetic gas theory collision rate coefficient between IA and X; *k*_evap_ and *k*_coag_ are the evaporation rate coefficient and coagulation sink rate coefficient of the (IA)_1_(X)_1_ dimer cluster, respectively; [X] and [IA] are the atmospheric concentrations of the X and IA, respectively; *P*_ref_ is the reference pressure at which Δ*G* is calculated (101,325 Pa); *k*_b_ is the Boltzmann constant; *T* is the temperature; Δ*G* is the calculated binding free energy of the (IA)_1_(X)_1_ dimer cluster; *r*_*i*_ are the radii of the monomers, which are presented in Table S1; μ is the reduced mass, which is equal to *m*_IA_*m*_X_/(*m*_IA_ + *m*_X_), where *m* is the mass of the monomers.

The atmospheric concentrations of precursors used here were derived from reported values from field observations (Table S1). It should be noted that the particle phase concentration was used instead when the gaseous concentration of a precursor was not found in the literature. In this case, the value may overestimate its gaseous concentrations and should be considered as an upper limit. The [(IA)_1_(HIO_2_)_1_] was selected as a reference point to assess the relative enhancing potential of other precursors, as HIO_2_ has previously been shown to be the highest enhancer in iodine oxoacid-induced nucleation in polar and marine environments.^14,18^ We evaluated the dimer cluster concentrations at 298.15 K with the [IA] and [HIO_2_] of 1 × 10^7^ and 3.33 × 10^5^ cm^–3^, respectively, based on their frequently observed concentrations in marine regions.^14^ In addition, a lower temperature (278.15 K) or lower concentrations of [IA] (3 × 10^6^ cm^–3^) and [HIO_2_] (1 × 10^5^ cm^–3^) were carried out to investigate the effects of temperature and concentrations of monomers on the formation of 63 dimer clusters. It should be noted that unless specified, similar to our latest study,^18^ an enhancement factor of 2.4 was used to correct the hard sphere collision rate coefficients based on the long-range transition state theory with dispersion force potential^44^ for all the studied systems in this study.

### QSAR Modeling

The calculated Δ*G* values of the 63 (IA)_1_(X)_1_ dimer clusters were used to construct a QSAR model by stepwise multiple linear regression. Similar to our previous studies,^25,45−50^ quantum chemical descriptors and Dragon molecular descriptors were considered for the modeling, which were calculated with the Gaussian 16 program^34^ and the Dragon 6.0 program,^51^ respectively. Dragon molecular descriptors were calculated based on the molecular structures optimized at the M06-2X/aug-cc-pVTZ(-PP) level of theory. The Δ*G* values of the 63 (IA)_1_(X)_1_ dimer clusters were randomly divided into training and validation sets with a ratio of 5:1. The determination coefficient (*R*^2^), root-mean-square error (RMSE), leave-one-out cross-validated (*Q*_LOO_^2^), 5-fold cross-validation (*Q*_*k*fold(*k*=5)_^2^), and external validation coefficient (*Q*_ext_^2^) were used to evaluate the goodness-of-fit, robustness, and predictive ability of the QSAR model. A Williams plot was used to characterize the applicability domain of the QSAR model. Additional details for QSAR model construction can be found in our previous studies.^25,47^

### Atmospheric Cluster Dynamics Code Modeling

The atmospheric cluster dynamics code (ACDC)^42^ was used to simulate the time evolution of the cluster formation rates, steady-state concentrations, and growth pathways of (IA)_*x*_(DEA)_*y*_ (*x* = 0–3, *y* = 0–3) clusters. Here, the simulation system was treated as a “3 × 3” box for the IA-DEA system, where 3 is the maximum number of IA or DEA molecules. The (IA)_4_(DEA)_3_ cluster was set as the boundary cluster and allowed to contribute to the cluster formation rate (see details in the Supporting Information). The [IA] and [DEA] were set to 10^5^, 10^6^, 10^7^, and 10^8^ cm^–3^,^14,15,52^ and 2.5 × 10^5^, 2.5 × 10^6^, 2.5 × 10^7^, 2.5 × 10^8^, and 5 × 10^8^ cm^–3^,^53−55^ respectively, corresponding to their atmospheric concentration ranges. Similar to a previous study,^21^ the setting values of [IA] are the initial concentrations of the IA monomer. The simulations were mainly run at 278.15 K with the *k*_coag_ of 2 × 10^–3^ s^–1^ (a typical value in coastal regions).^56^ To explore the temperature and *k*_coag_ effects on the IA-DEA nucleation, we additionally conducted simulations at other temperatures (263.15 and 283.15 K) and a large *k*_coag_ value (2 × 10^–2^ s^–1^, a value at coastal urban and polluted atmospheres^56^). In the simulations, the initial [IA] is redistributed to the [IA] and [(IA)_1_(DEA)_1–3_], and therefore the sum of the steady state [IA] and [(IA)_1_(DEA)_1–3_] equals to the initial [IA].

## Results and Discussion

### QSAR Modeling

Figures S2 and 1A show the identified global minimum structures and Δ*G* values of the 63 (IA)_1_(X)_1_ dimer clusters, respectively. By carefully checking the structures of all 63 dimer clusters, we found three types of interactions in the dimer clusters, including electrostatic interactions caused by proton transfer reactions, HBs, and XBs. While the (IA)_1_(HIO_2_)_1_ dimer cluster contains all three types of interactions, only two types of interactions can be synchronously formed at most in all other clusters. Among these three types of interactions, electrostatic interactions, HBs, and XBs are formed in 8, 53, and 44 dimer clusters, respectively. Therefore, XBs appear to be a common feature in the formation of IA-containing dimer clusters besides the HBs. This phenomenon differs the IA-containing dimer clusters from the SA/MSA-containing dimer clusters, where HBs are the most common interactions.^26−32,57−59^ Similar to the SA/MSA-containing dimer clusters, electrostatic interactions between positive and negative ions can only be formed in the dimer clusters with selected atmospheric bases (e.g., DMA, TMA, DEA, et al.). Only 8 bases among the considered 21 bases [including NH_3_, amines, and HIO_2_ (exhibiting a base behavior)^18,22^] can form electrostatic interactions with IA.

**Figure 1:**
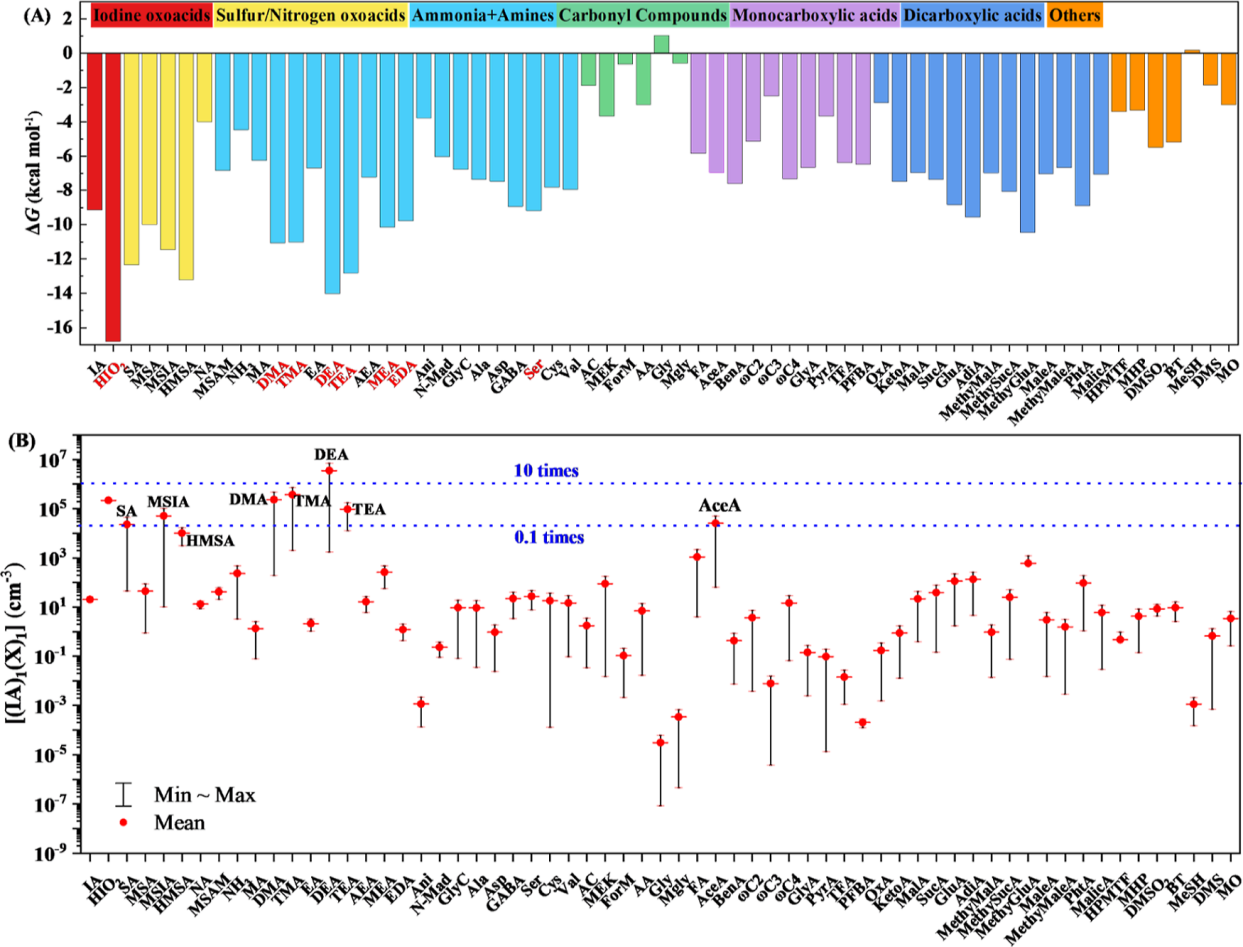
Calculated Δ*G* values of the 63 (IA)_1_(X)_1_ dimer clusters at the DLPNO-CCSD(T)//M06-2X/aug-cc-pVTZ(-PP) level of theory, 298.15 K, and 1 atm (A), and calculated [(IA)_1_(X)_1_] (cm^–3^) (B) at the condition of 298.15 K, *k*_coag_ = 2 × 10^–3^ s^–1^, [IA] = 1 × 10^7^ cm^–3^, and [HIO_2_] = 3.33 × 10^5^ cm^–3^. Names in red show clusters which exhibit proton transfer. The blue dashed lines show ratios of [(IA)_1_(X)_1_]/[(IA)_1_(HIO_2_)_1_].

As can be seen in Figure 1A, (IA)_1_(HIO_2_)_1_ has the lowest Δ*G* value (−16.79 kcal mol^–1^). Therefore, HIO_2_ has the strongest ability to bind with IA compared to the remaining 62 compounds. The involvement of all three interactions in the (IA)_1_(HIO_2_)_1_ dimer cluster should be the main reason for the strong stabilization, compared to all other dimer clusters. Besides (IA)_1_(HIO_2_)_1_, (IA)_1_(DEA)_1_ (−14.01 kcal mol^–1^), (IA)_1_(HMSA)_1_ (−13.21 kcal mol^–1^) (HMSA: hydroxymethanesulfonic acid), (IA)_1_(TEA)_1_ (−12.81 kcal mol^–1^) (TEA: triethylamine), and (IA)_1_(SA)_1_ (−12.34 kcal mol^–1^) have noticeably lower Δ*G* values than the other dimer clusters. Clusters with S-atom-containing acids, such as (IA)_1_(HMSA)_1_ and (IA)_1_(SA)_1_, have comparable binding free energies with clusters containing strong bases, such as (IA)_1_(DEA)_1_ and (IA)_1_(TEA)_1_, where acid–base reactions occur. The lower binding free energies of HMSA and SA with IA result from the formation of XBs in addition to the HBs. Some O-atom-containing organic acids, notably dicarboxylic acids, including 2-methylglutaric acid (MethyGluA, −11.45 kcal mol^–1^), adipic acid (AdiA, −9.55 kcal mol^–1^), phthalic acid (PhtA, −8.89 kcal mol^–1^), and glutaric acid (GluA, −8.83 kcal mol^–1^) have relatively lower Δ*G* values. Such lower Δ*G* values of the O-atom-containing organic acids also result from the formation of additional XBs. It should be noted that the Δ*G* value of MethyGluA is even lower than some of the atmospheric bases [i.e., MEA (ethanolamine, −10.15 kcal mol^–1^), DMA (−11.06 kcal mol^–1^), and TMA (−11.02 kcal mol^–1^)]. Differing from these O-atom-containing organic acids, carbonyl-containing compounds and other organic compounds, including hydroperoxymethyl thioformate (HPMTF), methyl hydroperoxide (MHP), dimethyl sulfone (DMSO_2_), benzothiazole (BT), methanethiol (MeSH), dimethyl sulfide (DMS), and methanol (MO) have substantially higher Δ*G* values.

Based on the calculated Δ*G* values, the following QSAR model for Δ*G* prediction was constructed at 298.15 K
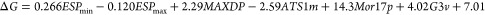

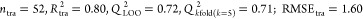

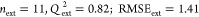
where *n*_tra_ and *n*_ext_ represent the number of the endpoint values in the training and validation data sets, respectively. This model contains six descriptors and their definition together with values are listed in Tables S2 and S3, respectively. According to the *t* values (Table S2), the Dragon descriptor *MAXDP* is the most significant contributor to the Δ*G* values. *MAXDP* refers to the maximal electrotopological positive variation, which is related to the electrophilicity of the molecule. In addition, some other Dragon descriptors, including *G3v*, *ATS1m,* and *Mor17p,* involved in the QSAR model are all related to the molecular structure. Furthermore, two quantum descriptors, *ESP*_min_ and *ESP*_max_, obtained from Multiwfn version 3.8^60^ are included in the QSAR. *ESP*_min_ and *ESP*_max_ refer to the global surface minimum and maximum of electrostatic potential, respectively. Both of them are useful for predicting intermolecular interactions. It has been reported that *ESP*_min_ correlates well with the interaction energy.^61^ The values of the statistical parameters (*R*^2^, *Q*_LOO_^2^, *Q*_*k*fold_^2^, and *Q*_ext_^2^) indicate that this QSAR model has high robustness, fitting goodness, and predictive abilities.

**Figure 2:**
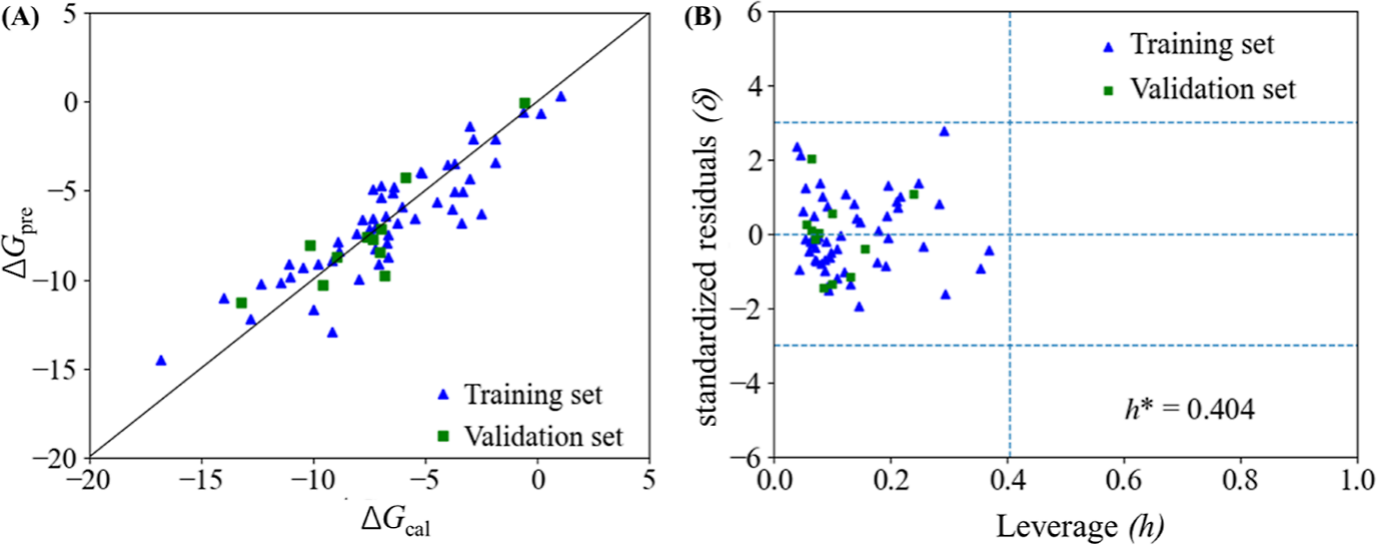
Predicted Δ*G* values (Δ*G*_pre_) from QSAR versus the calculated ones (Δ*G*_cal_) for all (IA)_1_(X)_1_ dimer clusters in the training and validation sets (A) and the Williams plot (B).

As shown in Table S2, the values of the variable inflation factors (VIF) for predictor variables are all in the range of 1–4, suggesting that there are no multiple correlations. From Figure 2A, it can be seen that the predicted Δ*G* values by the QSAR model match well with those calculated from the quantum chemical calculations. Moreover, the Williams plots (Figure 2B) demonstrate that the data set for the model is representative. Therefore, this QSAR model can offer an efficient way for high-throughput evaluation of the Δ*G* values of various compounds measured in the atmosphere with similar structures to the ones in the training set. According to the compounds utilized in developing the QSAR model, this model can be employed to predict the Δ*G* values of compounds with the following chemical formula: HIO_2–3_, RSO_2–4_, RSO_2_R′, RNO_3_, RNH_2_, R_2_NH, R_3_N, RCONH_2_, RCOOH, HOOCRCOOH, RC(O)H, RSH, RSR′, ROH, ROOH, RC=O, RS=OR′, R-ring-(C–S–C=N–C).

### Estimated Concentrations of the 63 (IA)_1_(X)_1_ Dimer Clusters

To further evaluate the enhancing potential of the 63 compounds on IA-induced nucleation, the atmospheric concentrations of the 63 (IA)_1_(X)_1_ dimer clusters were estimated based on 1. As shown in Figure 1B, the [(IA)_1_(X)_1_] has a great variation ranging from 10^–8^ to 10^6^ cm^–3^. Dimer clusters with mean concentrations higher than 10^5^ cm^–3^ include (IA)_1_(HIO_2_)_1_, (IA)_1_(DMA)_1_, (IA)_1_(TMA)_1_, and (IA)_1_(DEA)_1_. Despite (IA)_1_(HIO_2_)_1_ being the most stable dimer cluster, the [(IA)_1_(DEA)_1_] was found to be the highest (3.57 × 10^6^ cm^–3^), which is 16.5 times higher than that of the [(IA)_1_(HIO_2_)_1_]. This results from the much higher ambient concentration of DEA (about 10^5^–10^8^ cm^–3^) compared to HIO_2_ (about 10^3^–10^6^ cm^–3^). Therefore, DEA most likely has the highest enhancing potential for IA-induced nucleation among the 63 selected compounds. It should be noted that S-atom-containing acids [i.e., SA, MSIA (methanesulfinic acid), and HMSA] could also have a high enhancing potential for IA-induced nucleation based on their relatively higher dimer cluster concentrations (>10^4^ cm^–3^), which is consistent with the reported enhancing effect of SA on IA-induced nucleation.^38^ Additionally, we found that the dimer cluster concentrations of some of the O-atom-containing acids e.g., GluA, MethyGluA, and AdiA are at the ∼10^2^ cm^–3^ level, and AceA (acetic acid) is at the ∼ 10^4^ cm^–3^ level, also indicating their enhancing potential for IA-induced nucleation. It is known that HIO_2_ always co-exists with IA,^14,18^ thus iodine oxoacid (IA-HIO_2_) nucleation is the baseline of IA-induced nucleation. After considering this baseline, the compounds with the potential to enhance IA dimer cluster formation include DMA, TMA, TEA, DEA, SA, MSIA, HMSA, and AceA. However, as we only consider the average atmospheric conditions in this study, specific regions with higher precursor concentrations could yield different results and should be discussed separately.

It is well known that DMA has a high enhancing potential for SA-induced nucleation.^62,63^ Very recently, it was also found that DMA can enhance IA-induced nucleation.^21^ Interestingly, in this study, we find that the (IA)_1_(DEA)_1_ dimer cluster is much more stable than the (IA)_1_(DMA)_1_ dimer cluster, and the predicted [(IA)_1_(DEA)_1_] is higher than the [(IA)_1_(DMA)_1_] after accounting for the atmospheric concentrations of DEA ([DEA]) and DMA ([DMA]). In fact, the mean value of [DEA] is lower than that of [DMA] in the atmosphere (see Table S1). It is therefore the lower binding free energy (−14.01 kcal mol^–1^) of DEA with IA, compared to that of DMA (−11.06 kcal mol^–1^) with IA, that leads to the higher [(IA)_1_(DEA)_1_]. Therefore, DEA may have a higher enhancing potential than DMA for IA-induced nucleation. Our conclusion holds even when lower values of [IA] (3 × 10^6^ cm^–3^) and [HIO_2_] (1 × 10^5^ cm^–3^) are considered or at a lower temperature (278.15 K) (see Figures S3 and S4). Therefore, we further investigate the properties of larger IA-DEA clusters to evaluate the role of DEA in IA-induced nucleation.

### IA-DEA Cluster Structures

**Figure 3:**
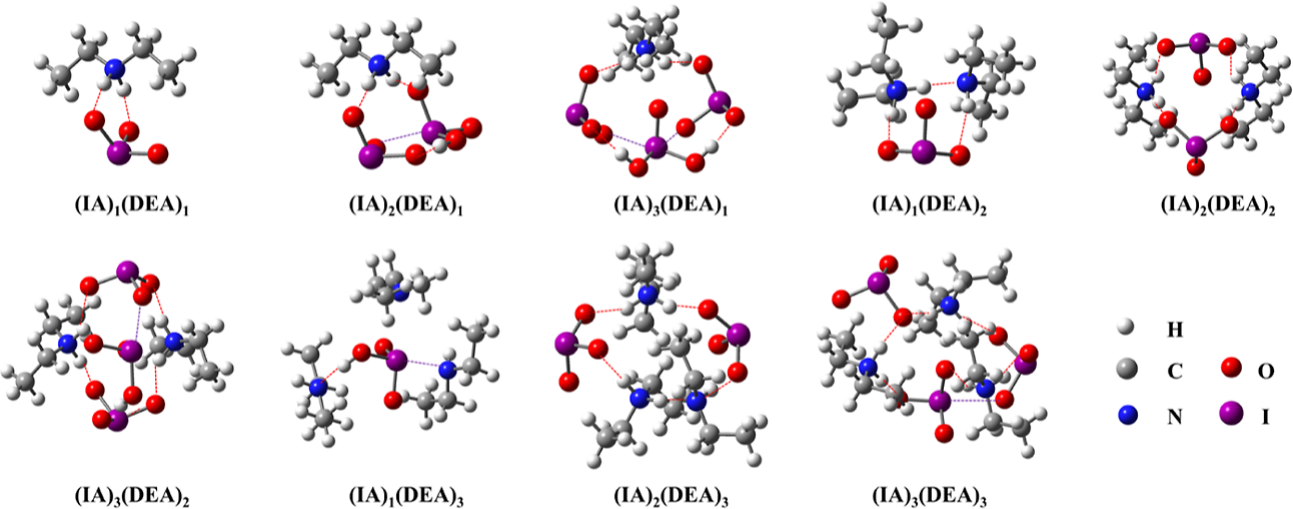
Identified global minimum configurations of the (IA)_*x*_(DEA)_*y*_ clusters (*x* = 1–3, *y* = 1–3) at the DLPNO-CCSD(T)//M06-2X/aug-cc-pVTZ(-PP) level of theory. The dashed red and purple lines indicate HBs and XBs, respectively.

Since previous studies have discussed the structures of (IA)_1–3_ and (DEA)_1–3_ clusters separately,^37,38^ here we mainly focus on the structures of (IA)_*x*_(DEA)_*y*_ clusters (*x* = 1–3, *y* = 1–3). The global minimum structures of (IA)_*x*_(DEA)_*y*_ clusters (*x* = 1–3, *y* = 1–3) are presented in Figure 3. We find that proton transfer reaction occurs in essentially all heteromolecular IA-DEA clusters except for the (IA)_1_(DEA)_3_ cluster. The (IA)_1_(DEA)_3_ cluster is stabilized by both HBs and XBs, and the rest are generally stabilized by a mixture of electrostatic interactions and HBs and/or XBs. It should be noted that we also located a (IA)_1_(DEA)_3_ cluster with proton transfer, but it has a 2.86 kcal mol^–1^ higher free energy compared to the identified lowest energy conformer with only HBs and XBs.

When *x* = *y* in (IA)_*x*_(DEA)_*y*_ clusters, each IA molecule transfers a proton to a DEA molecule, and all clusters are stabilized by electrostatic interactions as well as HBs. When *x* – *y* = 1 or 2, i.e., the (IA)_2_(DEA)_1_, (IA)_3_(DEA)_2_, and (IA)_3_(DEA)_1_ clusters, all DEA molecules are fully protonated, and the IA molecule that does not undergo proton transfer can form XBs with another IA molecule. Therefore, these clusters are stabilized by electrostatic interactions, HBs, and XBs. Interestingly, we find a spontaneous proton transfer reaction among the IA molecules in the (IA)_3_(DEA)_1_ cluster. The spontaneous proton transfer was confirmed by re-optimizing the “proton-returned” conformer, similar to our previous study.^18^ To the best of our knowledge, it is the first time that proton transfer reactions are observed between IA molecules, but a similar effect of atmospheric acids acting as bases has previously been observed for phosphoric acid^64^ and HIO_2_.^18,22^ When *y* – *x* = 1, such as the (IA)_1_(DEA)_2_ and (IA)_2_(DEA)_3_ clusters, one DEA molecule remains unprotonated. The unprotonated DEA molecule can bind with protonated DEA and IA molecules via HBs, leading to clusters stabilized by electrostatic interactions and HBs. Therefore, only the clusters with IA in equal amounts or in excess are stabilized by three types of interactions, i.e., electrostatic interactions, HBs, and XBs. The remaining clusters are primarily stabilized by two types of interactions, i.e., electrostatic interactions and HBs or HBs and XBs.

### Cluster Stability

**Figure 4:**
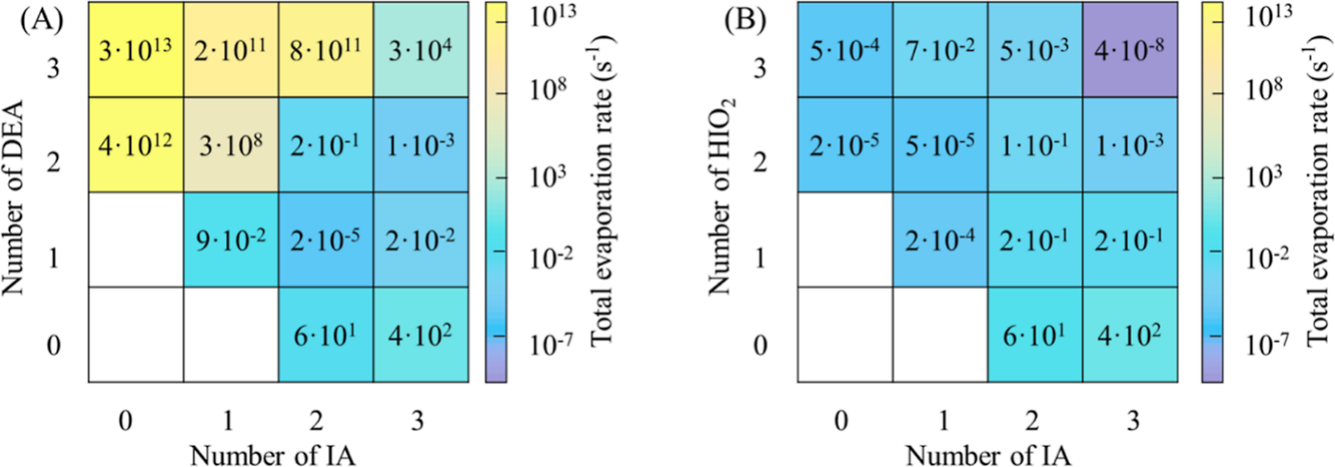
Evaporation rates of the (IA)_*x*_(DEA)_*y*_ (A) and (IA)_*x*_(HIO_2_)_*y*_ (original data adopted from Zhang et al.^18^) (B) clusters (*x* = 0–3, *y* = 0–3) at 278.15 K and 1 atm.

To further evaluate the stability of the IA-DEA clusters, evaporation rates (Figure 4) were calculated at 278.15 K based on the calculated Δ*G* values (Figure S5). In addition, the calculated Δ*G* values at 298.15 K are shown in Figure S6. In general, a lower evaporation rate translates into a higher cluster stability. As shown in Figure 4, the evaporation rates of DEA-abundant clusters (lying above the diagonal line) are greater than 10^8^ s^–1^, indicating that they will rapidly evaporate. The evaporation rates of the clusters with one more IA molecules than DEA molecules are lower than 10^–2^ s^–1^ (lying below the diagonal line) and thereby stable against evaporation. For the clusters with an equal number of DEA and IA (lying on the diagonal line), the smaller clusters, i.e., (IA)_1_(DEA)_1_ and (IA)_2_(DEA)_2_ are stable with evaporation rates of about 10^–2^ and 10^–1^ s^–1^, respectively, and the largest cluster (IA)_3_(DEA)_3_ is unstable with an evaporation rate of about 10^4^ s^–1^. The greater stability of IA-abundant heteromolecular clusters could result from the fact that the IA-abundant clusters include a variety of intermolecular interactions compared to other clusters, as discussed above. In addition, all pure IA and DEA clusters are unstable. The distribution of stable clusters of the IA-DEA system is similar to the case of the IA-DMA system^21^ and different from most of the SA-amine systems^27−29,57^ and the IA-HIO_2_ system.^18^

### Cluster Growth Pathway

**Figure 5:**
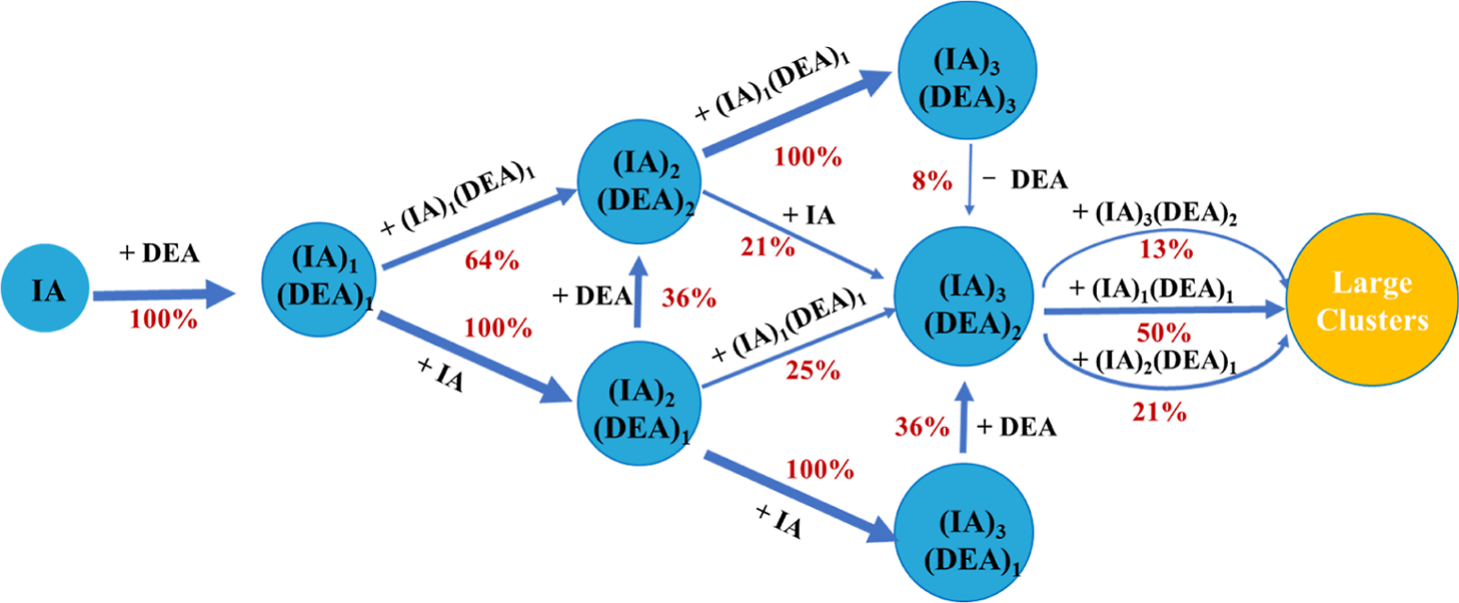
Cluster formation pathways for the IA-DEA system at 278.15 K, [IA] = 10^7^ cm^–3^, [DEA] = 2.5 × 10^7^ cm^–3^, and *k*_coag_ = 2.0 × 10^–3^ s^–1^.

Figure 5 shows the cluster growth pathways of the IA-DEA clusters at 278.15 K with [IA] = 10^7^ cm^–3^, [DEA] = 2.5 × 10^7^ cm^–3^, and *k*_coag_ = 2.0 × 10^–3^ s^–1^. The first step of the IA-DEA system is the formation of the (IA)_1_(DEA)_1_ dimer cluster, which is similar to the SA/MSA-amines and IA-DMA systems.^21,25−29,37,57,59^ The formed (IA)_1_(DEA)_1_ dimer cluster has two major growth pathways: (1) collision with an additional IA molecule to form the (IA)_2_(DEA)_1_ cluster; and (2) collision with the (IA)_1_(DEA)_1_ dimer cluster to form the (IA)_2_(DEA)_2_ cluster. The (IA)_2_(DEA)_1_ cluster can grow further to form the (IA)_2_(DEA)_2_ cluster. Both the (IA)_2_(DEA)_1_ and (IA)_2_(DEA)_2_ clusters have two growth pathways, which eventually lead to the formation of the (IA)_3_(DEA)_2_ cluster. The first pathway is a one-step pathway, i.e., (IA)_2_(DEA)_1_ collides with the (IA)_1_(DEA)_1_ dimer cluster and (IA)_2_(DEA)_2_ with one IA molecule. The other pathway is a two-step pathway, i.e., (IA)_2_(DEA)_1_ first collides with one IA molecule, followed by the addition of one DEA molecule. Alternatively, the (IA)_3_(DEA)_2_ cluster can be formed by the (IA)_2_(DEA)_2_ cluster colliding with the (IA)_1_(DEA)_1_ cluster, followed by the evaporation of a DEA molecule. Therefore, besides monomer condensation, cluster coagulation between the (IA)_1_(DEA)_1_ cluster and other clusters also plays an important role in the IA-DEA cluster growth. This results from the high stability of the (IA)_1_(DEA)_1_ cluster. Similar phenomenon of the involvement of small cluster in cluster growth was also found in the SA-DMA, IA-HIO_2_, and MSA/SA-guanidine systems.^18,25,42,57^ Among all clusters growing out of the 3 × 3 box, the (IA)_4_(DEA)_3_ cluster (50%) is the majority, followed by the (IA)_5_(DEA)_3_ (21%), (IA)_6_(DEA)_4_ (13%), and others (16%). In addition, it was found that the selection of the *k*_coag_ and temperature have little effect on the primary growth pathways for the IA-DEA system (Figure S7).

### Nucleation Rate Enhancement

**Figure 6:**
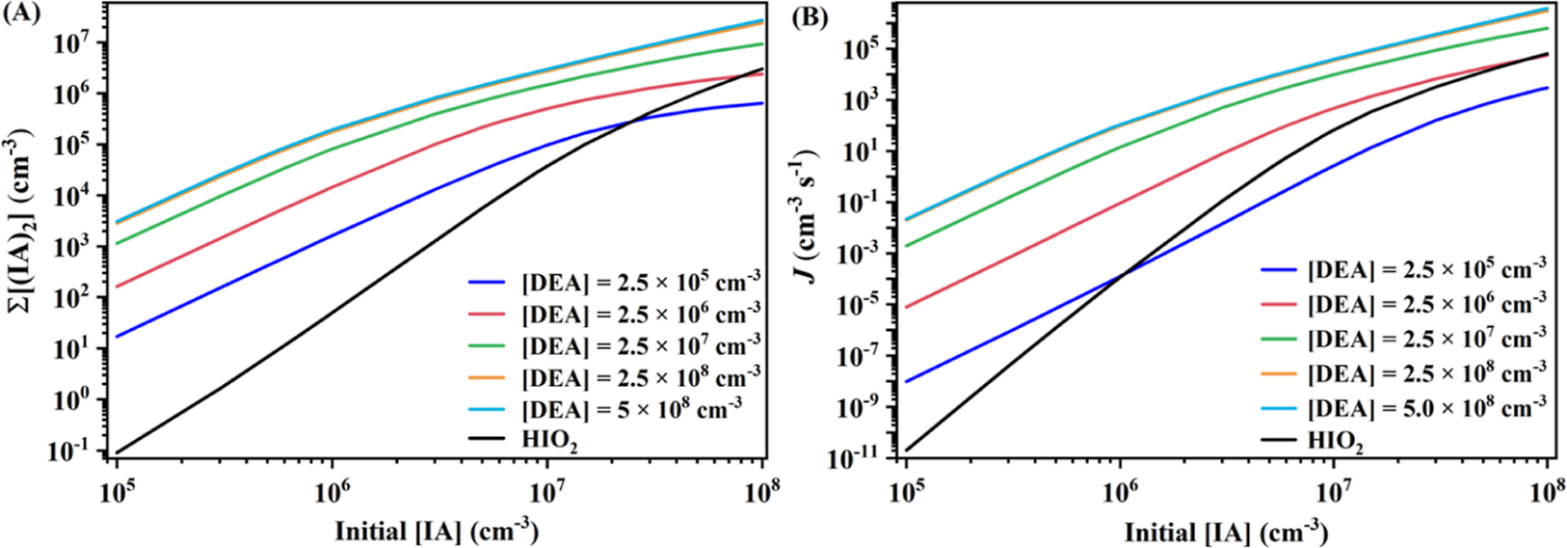
Simulated steady-state IA dimer cluster concentrations (∑[(IA)_2_] (cm^–3^) (A) and cluster formation rates *J* (cm^–3^ s^–1^) of the systems as a function of [DEA] or [HIO_2_] at 278.15 K, 1 atm, and *k*_coag_ = 2.0 × 10^–3^ s^–1^ (B).

Similar to our previous studies,^25,27−29^ the steady-state IA dimer concentration (∑[(IA)_2_]) and cluster formation rates (*J*) were calculated to evaluate the enhancing potential of DEA on IA-induced nucleation. The simulated ∑[(IA)_2_] and *J* as a function of initial [IA] (10^5^–10^8^ cm^–3^) and [DEA] (2.5 × 10^5^–5 × 10^8^ cm^–3^) for the IA-DEA system at *T* = 278.15 K, 1 atm, and *k*_coag_ = 2.0 × 10^–3^ s^–1^, are presented in Figure 6, along with the values of the IA-HIO_2_ system as a comparison. Additionally, the steady-state concentrations of IA monomer ([IA]) and heterodimer clusters ([(IA)_1_(DEA)_1_] and [(IA)_1_(HIO_2_)_1_] for the IA-DEA and IA-HIO_2_ systems are presented in Table S4. It should be noted that the ratio of [HIO_2_] to [IA] is set as 1:30, a common value observed in the CLOUD experiments.^14^ With the increase of [IA] and [DEA], both the ∑[(IA)_2_] and *J* gradually increase, and the curve flattens when [DEA] is greater than 2.5 × 10^8^ cm^–3^. We find that if the [DEA] ≥ 2.5 × 10^6^ cm^–3^ [0.1 parts per trillion (ppt)], the *J* value of the IA-DEA system is higher than or comparable to the corresponding value of the IA-HIO_2_ system (Figure 6B). The latter is believed to be the most effective IA-induced nucleation mechanism by the CLOUD experiments and our previous calculations.^14,18^ In addition, the ∑[(IA)_2_] of the IA-DEA system is higher than the IA-HIO_2_ system at [IA] < 10^7^ cm^–3^ and [DEA] = 2.5 × 10^6^ cm^–3^. At [DEA] beyond 2.5 × 10^6^ cm^–3^, both the ∑[(IA)_2_] and *J* values of the IA-DEA system are much higher than the corresponding values of the IA-HIO_2_ system. Moreover, at low [IA] (about 10^6^ cm^–3^) conditions, a higher [DEA] [≥2.5 × 10^7^ cm^–3^ (1 ppt)] can lead to notable *J* values. Therefore, DEA could be the strongest enhancing agent for IA-induced nucleation in DEA-rich environments at 278.15 K. Additionally, the revealed trend remains at other temperatures (263.15 and 283.15 K) and a larger *k*_coag_ value (2.0 × 10^–2^ s^–1^) (Figure S8).

### Atmospheric Implication

In this study, the Δ*G* values for 63 (IA)_1_(X)_1_ dimer clusters were calculated. The Δ*G* values of dimer clusters provide critical information on the enhancing potential of atmospheric nucleation precursors on IA-induced nucleation. This study reveals the important contribution of XBs to the Δ*G* values, distinct from the SA/MSA-precursor systems.^25,27−31,57,65−72^ This is the first evaluation of the enhancing potential of such a large number of precursors for IA-induced nucleation, expanding the current understanding of IA-induced nucleation. Furthermore, we developed a QSAR model to predict the Δ*G* values of other (IA)_1_(X)_1_ dimer clusters based on the 63 calculated Δ*G* values. This QSAR model provides a foundation for the future evaluation of the collective contribution of all potential atmospheric nucleation precursors within the model’s applicability domain for the IA-induced nucleation.

It was found that DEA has the highest potential to enhance IA-induced nucleation. The [DEA] > 2.5 × 10^6^ cm^–3^ (0.1 ppt) can reach an enhancement comparable to HIO_2_ in IA-induced nucleation. The detected atmospheric [DEA] and [IA] are higher than 2.5 × 10^6^ and 10^6^ cm^–3^, respectively, e.g., in the East Mediterranean^53^ and at Mace Head,^14^ where the maximum values of [DEA] and [IA] are 9.4 × 10^8^ cm^–3^ (37.5 ppt) and 10^8^ cm^–3^, respectively. A recent study based on long-term observations found that [IA] can reach about 2.8 × 10^6^ cm^–3^ in the polluted urban atmosphere in Beijing and Nanjing.^23^ As shown in Figure 6B, DEA with a ppt level can lead to notable *J* values (>10 cm^–3^ s^–1^) when [IA] is about 10^6^ cm^–3^. In fact, DEA at ppt levels has been detected in polluted urban atmospheres, including Beijing.^73^ Importantly, [DEA] is enhanced in polluted urban atmospheres where the ethanol-fueled vehicles are widely used.^73^ These findings suggest that the IA-DEA nucleation mechanism may play a role in aerosol nucleation in highly polluted urban atmospheres with high levels of [DEA] and [IA]. Therefore, the ambient relevance of the role of DEA in IA-induced nucleation warrants future studies.

This study reveals that IA can strongly bind with S-atom-containing acids (e.g., SA, MSIA, and HMSA) and certain O-atom-containing organic acids (OAs) (e.g., GluA, MethyGluA, AdiA, and AceA) besides amines and HIO_2_. It should be noted that S-atom-containing acids also bind strongly with amines and O-atom-containing OAs.^57,74−77^ Since iodine oxoacids, S-atom-containing acids, O-atom-containing OAs, and amines coexist in the polluted atmosphere,^23^ the synergistic nucleation of multi-components (e.g., ternary IA-SA/HMSA/MSIA-HIO_2_/OAs/amines, quaternary IA-HIO_2_-SA/HMSA/MSIA-OAs, IA-HIO_2_-SA/HMSA/MSIA-amines, etc.) may occur. Therefore, iodine oxoacid-containing multi-component nucleation should be studied in the future.

## Supporting Information Available

Global minimum of the 63 monomers; structures of dimer clusters; values and description of molecular descriptor; atmospheric concentration of monomers; radius of the selected 63 monomers; selection of boundary clusters; effects of temperature and [IA] on [(IA)_1_(X)_1_]; Δ*G* values of (IA)_*x*_(DEA)_*y*_ clusters at 278.15 and 298.15 K; steady state concentrations of IA monomer ([IA]) and heterodimer clusters ([(IA)_1_(DEA)_1_] and [(IA)_1_(HIO_2_)_1_] for the IA-DEA and IA-HIO_2_ systems in the ACDC simulations; effects of *k*_coag_ values and temperatures on the results for the IA-DEA system; and coordinates of all optimized IA-DEA clusters (PDF)
