# Supplementary material for: Enhancement of Atmospheric Nucleation Precursors on Iodic Acid-Induced Nucleation: Predictive Model and Mechanism
Source: Environ Sci Technol. 2023 Apr 21;57(17):6944–54. doi: 10.1021/acs.est.3c01034 (PMC10157892; doi:10.1021/acs.est.3c01034)
Supplement: Supplementary file 1 — es3c01034_si_001.pdf [file es3c01034_si_001.pdf]

Supporting Information

Enhancement of Atmospheric Nucleation Precursors on Iodic Acid-induced Nucleation: Predictive Model and Mechanism

Fangfang Ma[1], Hong-Bin Xie[1]*, Rongjie Zhang[1], Lihao Su[1], Qi Jiang[1], Weihao Tang[2], Jingwen Chen[1], Morten Engsvang[3], Jonas Elm[3], and Xu-Cheng He[4,5*]

[1]Key Laboratory of Industrial Ecology and Environmental Engineering (Ministry of Education), School of Environmental Science and Technology, Dalian University of Technology, Dalian 116024, China

[2]National-Regional Joint Engineering Research Center for Soil Pollution Control and Remediation in South China, Guangdong Key Laboratory of Integrated Agro-environmental Pollution Control and Management, Institute of Eco-environmental and Soil Sciences, Guangdong Academy of Sciences, Guangzhou 510650, China

[3]Department of Chemistry and iClimate, Aarhus University, Langelandsgade 140, DK-8000 Aarhus C, Denmark

[4]Institute for Atmospheric and Earth System Research/Physics, University of Helsinki, Helsinki, 00014, Finland

[5]Finnish Meteorological Institute, Helsinki, 00560, Finland

Totally, 36 pages, 8 figures and 4 tables

Supporting Information

Contents

Selection of Boundary Clusters....S3

Table S1. ....S4

Table S2. ....S7

Table S3. ....S8

Table S4. ....S10

Figure S1. ....S12

Figure S2. ....S15

Figure S3. ....S15

Figure S4. ....S15

Figure S5. ....S16

Figure S6. ....S16

Figure S7. ....S17

Figure S8. ....S18

Coordinates of all optimized IA-DEA clusters S19

References....S32

Selection of Boundary Clusters. As discussed in our previous studies, the boundary clusters are ones allowed to flux out the simulation box for further growth, therefore, these clusters are required to have favorable compositions for high stability (low evaporation rate). As can be seen in Figure 4, the IA-DEA clusters lying below the diagonal line have relatively low evaporation rates (in the range of 10^{-2} ~ 10^{-5} s^{-1}). Especially for $(IA)_x(DEA)_{x-1}$ clusters have evaporation rate less than 10^{-3} s^{-1} and therefore can be deemed as stable clusters. Therefore, we selected $(IA)_4(DEA)_3$ cluster as the boundary cluster in the ACDC simulation.

Table S1. Atmospheric concentrations (cm^{-3}) and radius (Å) of 63 compounds detected in the marine regions.

Compounds	Name	CAS	Radius	Concentration range	Ref.
IA	Iodic Acid	7782-68-5	2.47[b]	$10^{5} \sim 10^{8}$	1,2
HIO_2	Iodous Acid	12134-99-5	2.88[c]	$3.33 \times 10^{3} \sim 3.33 \times 10^{6}$	2
SA	Sulfphuric acid	7664-93-9	2.77[b]	$10^{5} \sim 10^{8}$	3,4
MSA	Sulfonic acid	75-75-2	2.95[b]	$10^{5} \sim 10^{7}$	5,6
MSIA	Methanesulfinic acid	17696-73-0	2.99[c]	$10^{5} \sim 10^{9}$	7
HMSA	Hydroxymethanesulfonic Acid	75-92-3	3.45[c]	$1.61 \times 10^{6} \sim 8.59 \times 10^{6}$	8
NA	Nitric acid	7697-37-2	2.55[b]	$2.50 \times 10^{10} \sim 5.00 \times 10^{10}$	9
MSAM	Methanesulfonamide	3144-09-0	3.22[c]	$5.00 \times 10^{8} \sim 1.50 \times 10^{9}$	10
NH_3	Ammonia	7664-41-7	2.13[b]	$4.24 \times 10^{9} \sim 6.00 \times 10^{11}$	11,12
MA	Methylamine	74-89-5	2.66[b]	$5.00 \times 10^{6} \sim 1.65 \times 10^{8}$	13,14
DMA	Dimethylamine	124-40-3	2.97[b]	$3.61 \times 10^{6} \sim 8.91 \times 10^{9}$	15,16
TMA	Trimethylamine	75-50-3	3.27[b]	$4.07 \times 10^{7} \sim 1.50 \times 10^{10}$	17,18
EA	Ethanamine	75-04-7	2.96[b]	$3.14 \times 10^{7} \sim 9.73 \times 10^{7}$	19
DEA	Diethylamine	109-89-7	3.45[b]	$2.29 \times 10^{5} \sim 9.38 \times 10^{8}$	17,19
TEA	Triethylamine	121-44-8	3.81[b]	1.25×10^{7}[a] $\sim 1.78 \times 10^{8}$	14,17
AEA	Aminomethanol	3088-27-5	2.59[c]	$7.28 \times 10^{7} \sim 3.30 \times 10^{8}$	19
MEA	Ethanolamine	141-43-5	2.88[b]	$4.93 \times 10^{6} \sim 4.10 \times 10^{7}$	19
EDA	Ethylenediamine	107-15-3	2.98[b]	$7.22 \times 10^{4} \sim 3.43 \times 10^{5}$	19
Ani	Aniline	62-53-3	3.31[b]	$5.66 \times 10^{5} \sim 9.05 \times 10^{6}$	14,19
N-Mad	N-Methylformamide	123-39-7	2.87[b]	$8.19 \times 10^{6} \sim 3.44 \times 10^{7}$	19

GlyC	Glycine	56-40-6	2.95[b]	$2.17 \times 10^{6} \sim 5.07 \times 10^{8}$	19
Ala	Alanine	56-41-7	2.91[b]	$3.61 \times 10^{5} \sim 1.87 \times 10^{7}$	19,20
Asp	Aspartic acid	56-84-8	3.14[b]	$3.01 \times 10^{5} \sim 2.35 \times 10^{7}$	19,20
GABA	γ-aminobutyric acid	56-12-2	3.98[c]	$2.41 \times 10^{6} \sim 2.83 \times 10^{7}$	21
Ser	Serine	56-45-1	2.96[b]	$3.61 \times 10^{6} \sim 2.18 \times 10^{7}$	19,22
Cys	Cysteine	52-90-4	4.04[c]	$6.02 \times 10^{2} \sim 1.68 \times 10^{8}$	19,23
Val	Valine	72-18-4	3.36[b]	$3.61 \times 10^{5} \sim 1.09 \times 10^{8}$	19,22
AC	Acetone	67-64-1	3.08[b]	$3.50 \times 10^{9} \sim 3.62 \times 10^{11}$	24
MEK	2-Butanone	78-93-3	3.29[b]	$7.50 \times 10^{7} \sim 8.87 \times 10^{11}$	25
ForM	Formaldehyde	50-00-0	2.23[b]	$1.75 \times 10^{9} \sim 1.75 \times 10^{11}$	26
AA	Acetaldehyde	75-07-0	2.82[b]	$2.50 \times 10^{8} \sim 2.13 \times 10^{11}$	24,25
Gly	Glyoxal	107-22-2	2.72[b]	$1.14 \times 10^{6} \sim 8.59 \times 10^{8}$	27,28
Mgly	Methylglyoxal	78-98-8	3.01[b]	$4.18 \times 10^{6} \sim 6.20 \times 10^{8}$	16,28
FA	Formic Acid	64-18-6	2.46[b]	$5.00 \times 10^{8} \sim 2.75 \times 10^{11}$	29,30
AceA	Acetic acid	64-19-7	2.84[b]	$1.25 \times 10^{9} \sim 1.00 \times 10^{12}$	29,30
BenA	Benzoic Acid	65-85-0	3.34[b]	4.93×10^{4}[a] $\sim 5.62 \times 10^{6}$	31
ωC2	Glyoxylic acid	298-12-4	2.74[b]	$1.63 \times 10^{6} \sim 3.20 \times 10^{9}$	28,32
ωC3	3-Oxopropanoic acid	926-61-4	3.91[c]	$1.37 \times 10^{5} \sim 5.72 \times 10^{8}$	28,32
ωC4	4-Oxobutanoic acid	692-29-5	4.38[c]	$7.08 \times 10^{5} \sim 3.12 \times 10^{8}$	28,32
GlyA	Glycolic Acid	79-14-1	2.73[b]	$7.92 \times 10^{4} \sim 9.02 \times 10^{6}$	32
PyrA	Pyruvic acid	127-17-3	3.51[c]	$6.84 \times 10^{4} \sim 9.75 \times 10^{8}$	33,34
TFA	Trifluoroacetic Acid	76-05-1	3.09[b]	$5.81 \times 10^{4} \sim 1.43 \times 10^{6}$	35
PFBA	Heptafluorobutyric Acid	375-22-4	3.89[c]	$5.60 \times 10^{3} \sim 1.27 \times 10^{4}$	36

OxA	Oxalic acid	144-62-7	2.66[b]	$2.96 \times 10^7 \sim 6.55 \times 10^9$	[28,37]
KetoA	Ketomalonic acid	473-90-5	4.20[c]	$1.02 \times 10^5 \sim 1.40 \times 10^7$	[34,38]
MalA	Malonic acid	141-82-2	2.94[b]	$7.52 \times 10^6 \sim 8.33 \times 10^8$	[28,39]
SucA	Succinic acid	110-15-6	3.10[b]	$1.48 \times 10^6 \sim 7.70 \times 10^8$	[28,39]
GluA	Glutaric acid	110-94-1	3.34[b]	$1.46 \times 10^6 \sim 1.88 \times 10^8$	[28,32]
AdiA	Adipic acid	124-04-9	3.49[b]	$1.11 \times 10^6 \sim 6.38 \times 10^7$	[32,34]
MethyMalA	Methylmalonic acid	516-05-2	3.74[c]	$2.55 \times 10^5 \sim 3.52 \times 10^7$	[28,39]
MethySucA	Methylsuccinic acid	498-21-5	3.34[b]	$2.28\times 10^5 \sim 1.52 \times 10^8$	[28,34]
MethyGluA	2-Methylglutaric acid	617-62-9	5.01[c]	$0 \sim 6.43 \times 10^7$	[28]
MaleA	Maleic acid	110-16-7	3.07[b]	$2.59\times 10^5 \sim 1.03 \times 10^8$	[28,40,41]
MethyMaleA	Methylmaleic acid	498-23-7	4.40[c]	$1.11 \times 10^6 \sim 6.38 \times 10^7$	[28,40]
PhtA	Phthalic acid	88-99-3	3.46[b]	$7.97 \times 10^5 \sim 1.41 \times 10^8$	[32,34]
MalicA	Malic acid	6915-15-7	3.21[b]	$4.94 \times 10^5 \sim 1.93 \times 10^8$	[32]
HPMTF	Hydroperoxymethyl thioformate	-	3.78[c]	$0 \sim 7.50 \times 10^9$	[42]
MHP	Methyl hydroperoxide	3031-73-0	2.93[c]	$1.25 \times 10^9 \sim 7.50 \times 10^{10}$	[43]
$DMSO_2$	Dimethyl Sulfone	67-71-0	3.38[c]	$1.00 \times 10^9 \sim 3.00 \times 10^9$	[10]
BT	Benzothiazole	95-16-9	3.50[b]	$1.00 \times 10^9 \sim 6.30 \times 10^9$	[44]
MeSH	Methanethiol	74-93-1	2.71[b]	$5.00 \times 10^8 \sim 6.85 \times 10^9$	[44]
DMS	Dimethyl sulfide	75-18-3	3.07[b]	$7.50 \times 10^7 \sim 1.42 \times 10^{11}$	[24,44]
MO	Methanol	67-56-1	2.53[b]	$4.00 \times 10^9 \sim 1.00 \times 10^{11}$	[24]

[a]The detected limit values of TEA (0.5 ppt) and BenA (0.01 ng/cm^3) were selected as the minimum, respectively. [b]Calculated from the bulk liquid density of the monomers. [c]Calculated in Multiwfn (version 3.8)[45] by the maximum distance between two atoms considering their van der Waals radii.

Table S2. Definition and the corresponding *t*, *p*, and variable inflation factor (*VIF*) values of the involved descriptors in the QSAR model.

Descriptors	Depiction	*t*	*p*	*VIF*
ESP_{min}	Global surface minimum of electrostatic potential	5.53	< 0.001	1.12
ESP_{max}	Global surface maximum of electrostatic potential	-4.66	< 0.001	3.34
MAXDP	Maximal electrotopological positive variation (E-state indices)	6.02	< 0.001	2.33
ATS1m	Broto-Moreau autocorrelation of lag 1 (log function) weighted by mass	-5.16	< 0.001	1.71
Mor17p	signal 17/weighted by polarizability (3D-MoRSE descriptors)	4.22	< 0.001	1.72
G3v	3rd component symmetry directional WHIM index/weighted by van der Waals volume	5.31	< 0.001	1.24

Table S3. Values of descriptors used in the training set and validation set for the developed QSAR model.

Name	CAS	ESP_{min}	ESP_{max}	*MAXDP*	*ATS1m*	*Mor17p*	*G3v*
				Training set			
IA	7782-68-5	-30.4993	60.40009	1.684	3.769	0.011	0.301
HIO_2	12134-99-5	-39.0719	60.95545	1.596	3.376	-0.005	0.333
SA	7664-93-9	-25.8942	69.7153	1.743	2.738	0.004	0.263
MSA	75-75-2	-32.7486	62.51936	2.188	2.688	-0.102	0.24
MISA	17696-73-0	-37.6627	51.59789	2.111	2.411	-0.037	0.25
NA	7697-37-2	-19.0799	68.77906	1.361	1.753	0.014	1
NH_3	7664-41-7	-37.5833	26.52681	0	0.258	-0.011	0.333
MA	74-89-5	-36.4667	24.99615	0.5	0.961	-0.057	0.263
DMA	124-40-3	-34.1112	24.95134	0.25	1.37	-0.142	0.231
TMA	75-50-3	-31.5682	10.23146	0	1.659	-0.279	0.269
EA	75-04-7	-36.0518	24.75432	0.847	1.331	-0.126	0.231
DEA	109-89-7	-33.2141	22.99627	0.611	1.836	-0.283	0.242
TEA	121-44-8	-29.7283	11.05851	0.375	2.17	-0.433	0.238
AEA	3088-27-5	-32.0191	44.08632	1.347	1.38	-0.057	0.25
EDA	107-15-3	-38.6394	27.12002	0.903	1.622	-0.095	0.218
Ani	62-53-3	-25.8092	36.17895	1.358	2.173	-0.056	0.377
N-mad	123-39-7	-43.2885	47.73586	2.063	1.629	-0.011	1
GlyC	56-40-6	-31.1191	53.46982	2.243	1.842	-0.013	0.273
Asp	56-84-8	-31.8971	56.27833	2.846	2.413	-0.085	0.222
Ser	56-45-1	-33.8406	56.66515	2.645	2.179	-0.096	0.208
Cys	52-90-4	-26.5199	56.81051	2.756	2.331	-0.061	0.208
Val	72-18-4	-31.0337	52.23011	3.016	2.284	-0.294	0.191
AC	67-64-1	-37.3542	20.9595	2.444	1.576	-0.149	1
MEK	78-93-3	-37.2464	20.2537	2.813	1.793	-0.206	0.364
ForM	50-00-0	-30.4084	26.03983	1	0.916	-0.02	1
AA	75-07-0	-34.6089	22.42544	1.806	1.3	-0.087	1
Gly	107-22-2	-22.4871	32.50912	1.806	1.575	-0.058	1
AceA-	64-19-7	-33.886	50.95618	2	1.615	-0.069	1
ωC2	298-12-4	-29.6288	52.77662	2	1.823	-0.046	1
ωC3	926-61-4	-30.8492	58.80394	2.368	1.996	-0.029	0.53
ωC4	692-29-5	-31.1819	53.81022	2.599	2.143	-0.11	1

GlyA	79-14-1	-30.105	59.53015	2.118	1.855	-0.024	0.24
PyrA	127-17-3	-31.709	46.63017	2.544	1.996	-0.076	1
TFA	76-05-1	-23.9643	68.3402	2.579	2.254	0.025	0.588
PFBA	375-22-4	-23.4648	69.10038	3.751	2.882	0.031	0.208
OxA	144-62-7	-23.3124	55.26743	2.1	2.022	0.01	1
ketoA	473-90-5	-24.4857	63.59614	2.644	2.291	-0.076	0.231
MalA	141-82-2	-29.1445	56.888	2.429	2.166	0.007	0.543
SucA	110-15-6	-29.5148	53.6459	2.64	2.291	-0.031	0.259
GluA	110-94-1	-29.6771	51.82731	2.788	2.403	-0.155	0.197
MethysucA	498-21-5	-32.7168	51.34238	2.971	2.403	-0.071	0.217
MethygluA	617-62-9	-33.4614	53.06934	3.119	2.504	-0.188	0.25
MethyMaleA	498-23-7	-41.2833	65.58828	2.903	2.388	-0.093	1
PhtA	88-99-3	-31.4869	54.19327	3.463	2.701	-0.134	0.247
Malic	6915-15-7	-33.1978	61.95846	2.721	2.42	-0.052	0.228
HPMTF	-	-27.2374	49.53006	2.336	2.411	-0.083	0.357
MHP	3031-73-0	-28.7293	47.26144	1.069	1.498	-0.094	1
$DMSO_2$	67-71-0	-37.79	31.30681	2.632	2.636	-0.209	1
BT	95-16-9	-31.9352	25.75987	1.137	2.714	0.075	1
MeSH	74-93-1	-20.2769	21.00634	0.306	1.422	-0.052	1
DMS	75-18-3	-22.6789	15.14572	0.042	1.924	-0.117	1
MO	67-56-1	-33.4567	42.89407	1	0.992	-0.072	1

Name	CAS	ESP_{min}	ESP_{max}	*MAXDP*	*ATS1m*	*Mor17p*	*G3v*
Validation set							
benA	65-85-0	-32.9806	51.60094	3.201	2.416	-0.116	0.228
MEA	141-43-5	-38.3587	31.96722	1.75	1.638	-0.12	0.224
MethyMalA	516-05-2	-30.9697	55.39029	2.76	2.291	-0.033	0.208
MSAM	3144-09-0	-34.3064	43.95954	2.41	2.664	-0.132	0.231
MaleA	110-16-7	-40.9205	68.38905	2.554	2.274	-0.023	1
FA	64-18-6	-30.9032	55.85379	1.361	1.351	0.002	1
AdiA	124-04-9	-33.8834	51.44565	2.897	2.504	-0.221	0.219
HMSA	75-92-3	-28.9829	65.85052	2.306	2.777	-0.12	0.231
GABA	56-12-2	-33.5906	46.78989	2.703	2.157	-0.181	0.2
Ala	56-41-7	-31.2291	52.69487	2.574	2.012	-0.115	0.213
Mgly	78-98-8	-26.6108	29.35758	2.444	1.792	-0.099	1

Table S4. Steady state concentration (cm^{-3}) of IA monomer ([IA]) and heterodimer clusters ([$(IA)_1(DEA)_1$] and [$(IA)_1(HIO_2)_1$] for the IA-DEA and IA-HIO_2 systems in the ACDC simulations at 278.15 K, 1 atm and k_{coag} = 2.0 × 10^{-3} s^{-1}.

	IA-DEA system										IA-HIO_2 system	
	[DEA] = 2.5 ×10^5 (0.01 ppt)		[DEA] = 2.5 ×10^6 (0.1 ppt)		[DEA] = 2.5 ×10^7 (1 ppt)		[DEA] = 2.5 ×10^8 (10 ppt)		[DEA] = 5.0 ×10^8 (20 ppt)		-	
Initial [IA]	[IA]	[$(IA)_1(DEA)_1$]	[IA]	[$(IA)_1(DEA)_1$]	[IA]	[$(IA)_1(DEA)_1$]	[IA]	[$(IA)_1(DEA)_1$]	[IA]	[$(IA)_1(DEA)_1$]	[IA]	[$(IA)_1(HIO_2)_1$]
100000	99648	352	96588	3412	73899	26101	22070	77930	12404	87596	99905	95
300000	298946	1054	289786	10214	221838	78162	66368	233632	37315	262685	299147	848
500000	498247	1753	483011	16989	369974	130026	110898	389102	62376	437624	497628	2345
600000	597899	2101	579634	20366	444120	155880	133257	466743	74967	525033	596595	3360
900000	896858	3142	869546	30454	233120	233120	200733	699267	113000	787000	892362	7489
1000000	996513	3487	966198	33802	741245	258755	223363	776637	125767	874233	990590	9206
3000000	2989750	10250	2900728	99272	2240902	759098	691022	2308978	390998	2609002	2918114	76681
5000000	4983259	16741	4838125	161875	3763833	1236167	1187278	3812722	675219	4324781	4783986	193941
6000000	5980111	19889	5807878	192122	4533559	1466441	1445872	4554128	824368	5175632	5699602	264800
9000000	8971037	28963	8721143	278857	6873312	2126688	2262119	6737881	1299422	7700578	8399410	507644
10000000	9968130	31870	9693500	306500	7662783	2337217	2547313	7452687	1466823	8533177	9290668	593846
15000000	14954413	45587	14563906	436094	11674210	3325790	4066800	10933200	2369677	12630323	13725001	1040442
30000000	29920080	79920	29244378	755622	24216924	5783076	9462788	20537212	5693464	24306536	27161130	2336590
50000000	49885720	114280	48928904	1071096	41745935	8254065	18264967	31735033	11393571	38606428	45470587	3853557
60000000	59871956	128044	58803114	1196886	50743333	9256667	23226463	36773537	14719109	45280890	54763286	4516391
90000000	89839740	160260	88508646	1491354	78345344	11654656	39871806	50128194	26310777	63689223	82997667	6211695
100000000	99831227	168773	98430594	1569406	87695432	12304568	45908716	54091284	30648301	69351699	92510150	6688500

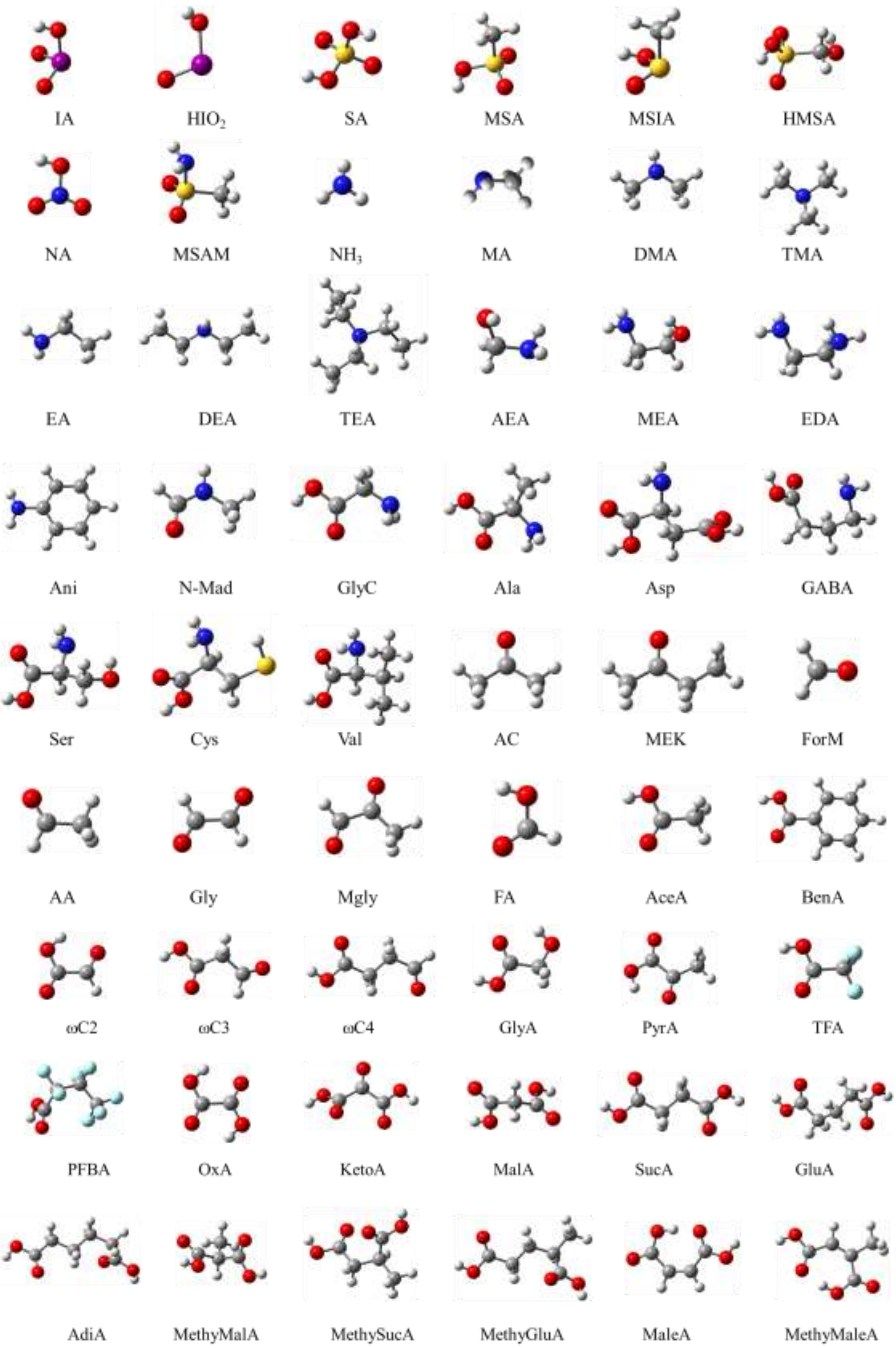
IA
HIO_2
SA
MSA
MSIA
HMSA
NA
MSAM
NH_3
MA
DMA
TMA
EA
DEA
TEA
AEA
MEA
EDA
Ani
N-Mad
GlyC
Ala
Asp
GABA
Ser
Cys
Val
AC
MEK
ForM
AA
Gly
Mgly
FA
AceA
BenA
ωC2
ωC3
ωC4
GlyA
PyrA
TFA
PFBA
OxA
KetoA
MalA
SucA
GluA
AdiA
MethyMalA
MethySucA
MethyGluA
MaleA
MethyMaleA

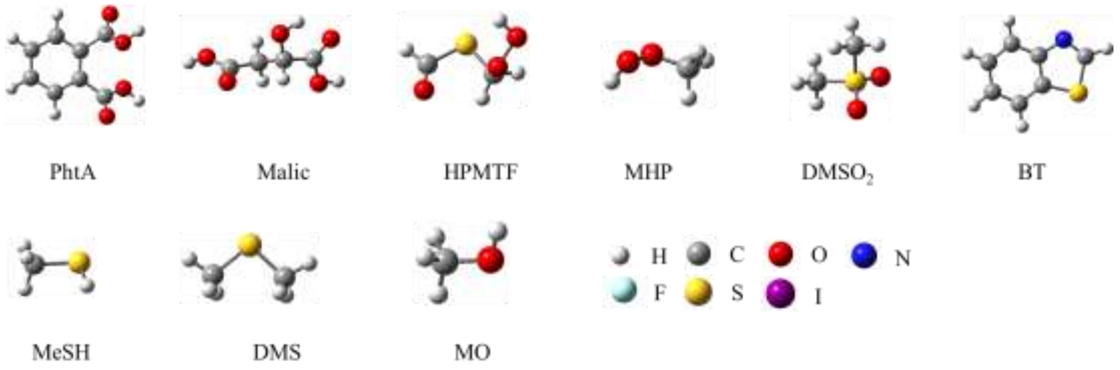

Figure S1. Global minimum configurations of selected 63 species.

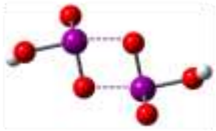
(IA)$_2$

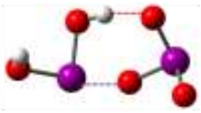
(IA)$_1$(HIO$_2$)$_1$

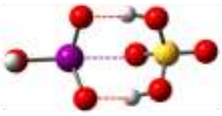
(IA)$_1$(SA)$_1$

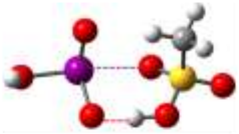
(IA)$_1$(MSA)$_1$

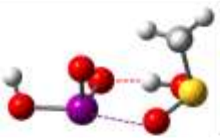
(IA)$_1$(MSIA)$_1$

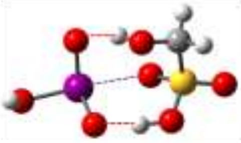
(IA)$_1$(HMSA)$_1$

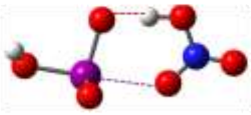
(IA)$_1$(NA)$_1$

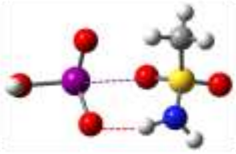
(IA)$_1$(MSAM)$_1$

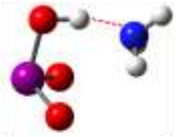
(IA)$_1$(NH$_3$)$_1$

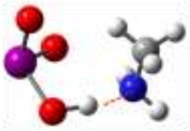
(IA)$_1$(MA)$_1$

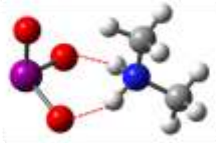
(IA)$_1$(DMA)$_1$

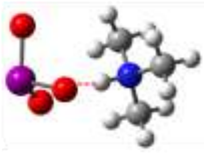
(IA)$_1$(TMA)$_1$

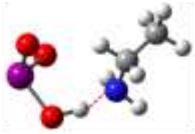
(IA)$_1$(EA)$_1$

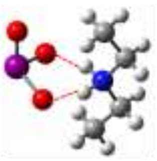
(IA)$_1$(DEA)$_1$

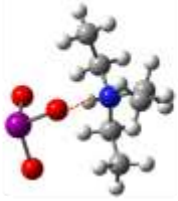
(IA)$_1$(TEA)$_1$

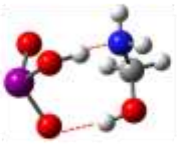
(IA)$_1$(AEA)$_1$

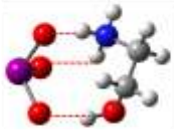
(IA)$_1$(MEA)$_1$

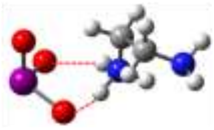
(IA)$_1$(EDA)$_1$

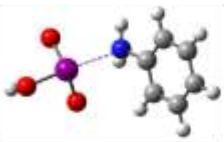
(IA)$_1$(Ani)$_1$

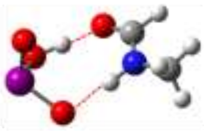
(IA)$_1$(N-Mad)$_1$

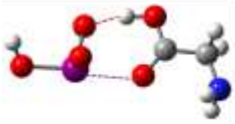
(IA)$_1$(GlyC)$_1$

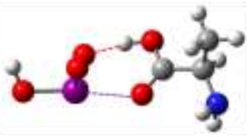
(IA)$_1$(Ala)$_1$

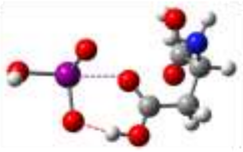
(IA)$_1$(Asp)$_1$

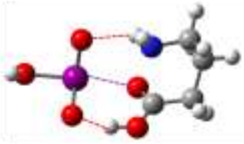
(IA)$_1$(GABA)$_1$

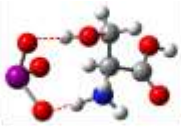
$(IA)_1(Ser)_1$

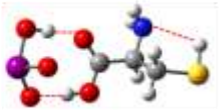
$(IA)_1(Cys)_1$

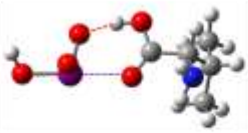
$(IA)_1(Val)_1$

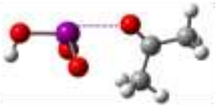
$(IA)_1(AC)_1$

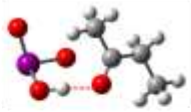
$(IA)_1(MEK)_1$

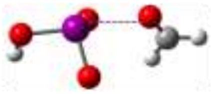
$(IA)_1(ForM)_1$

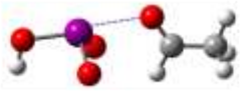
$(IA)_1(AA)_1$

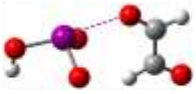
$(IA)_1(Gly)_1$

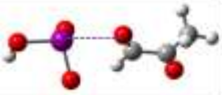
$(IA)_1(Mgly)_1$

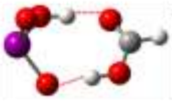
$(IA)_1(FA)_1$

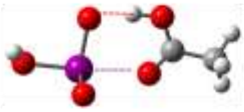
$(IA)_1(AceA)_1$

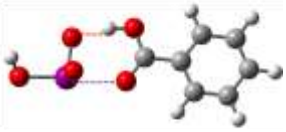
$(IA)_1(BenA)_1$

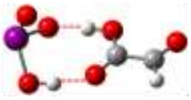
$(IA)_1(\omega C2)_1$

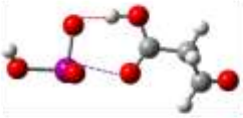
$(IA)_1(\omega C3)_1$

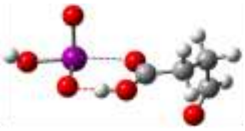
$(IA)_1(\omega C4)_1$

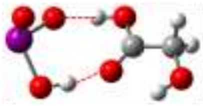
$(IA)_1(GlyA)_1$

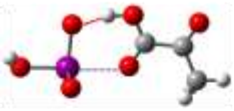
$(IA)_1(PyrA)_1$

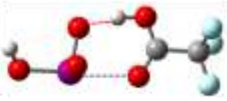
$(IA)_1(TFA)_1$

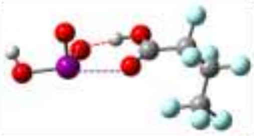
$(IA)_1(PFBA)_1$

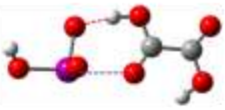
$(IA)_1(OxA)_1$

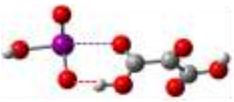
$(IA)_1(KetoA)_1$

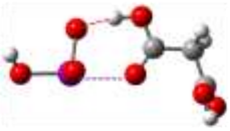
$(IA)_1(MalA)_1$

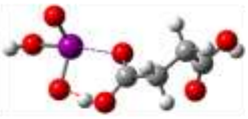
$(IA)_1(SucA)_1$

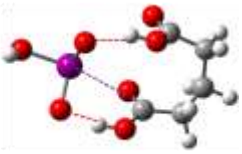
$(IA)_1(GluA)_1$

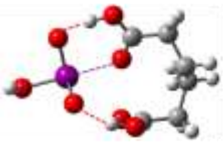
$(IA)_1(AdiA)_1$

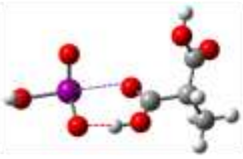
$(IA)_1(MethyMalA)_1$

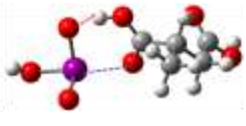
$(IA)_1(MethySucA)_1$

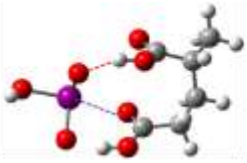
$(IA)_1(MethyGluA)_1$

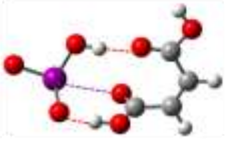
$(IA)_1(MaleA)_1$

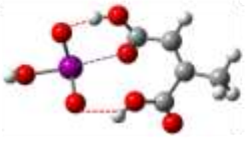
$(IA)_1(MethyMaleA)_1$

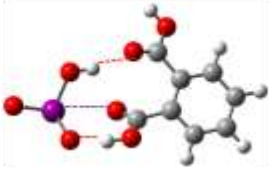
$(IA)_1(PhtA)_1$

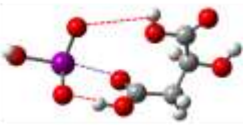
$(IA)_1(Malic)_1$

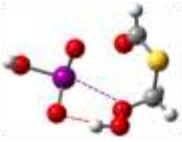
$(IA)_1(HPMTF)_1$

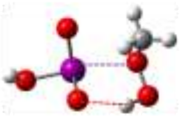
$(IA)_1(MHP)_1$

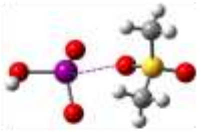
$(IA)_1(DMSO_2)_1$

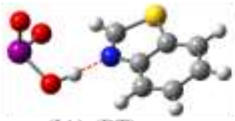
$(IA)_1(BT)_1$

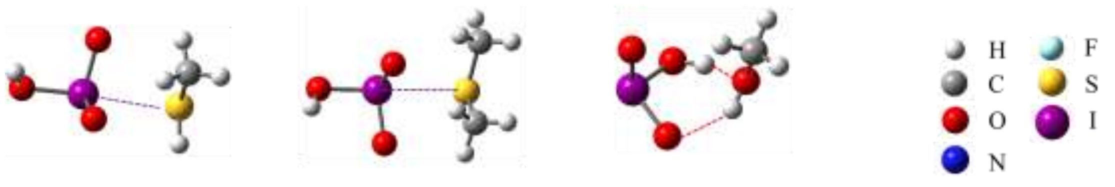

(IA)$_1$(MeSH)$_1$ (IA)$_1$(DMS)$_1$ (IA)$_1$(MO)$_1$

Figure S2. Lowest Gibbs free energy conformations of the 63 (IA)$_1$(X)$_1$ clusters at the DLPNO-CCSD(T)//M06-2X/aug-cc-pVTZ(-PP) level of theory. Dashed red and purple lines indicate hydrogen bonds and halogen bonds, respectively.

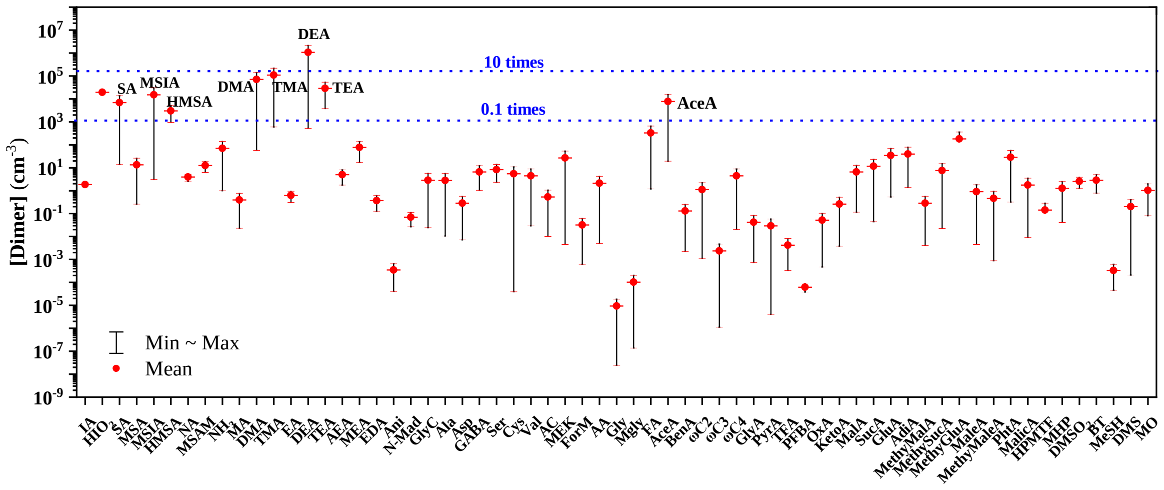

Figure S3. Calculated 63 [Dimer] (cm^{-3}) at the condition of 298.15 K, $k_{\mathrm{coag}} = 2.0 \times 10^{-3}$ s^{-1}, [IA] = 3 × 10^6 cm^{-3} and [HIO$_2$] = 1 × 10^5 cm^{-3}.

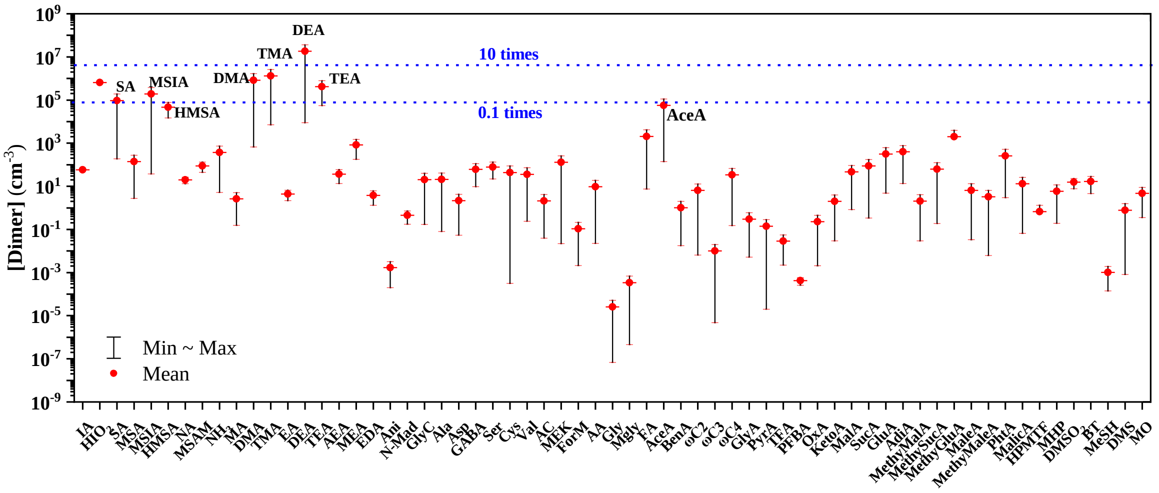

Figure S4. Calculated 63 [Dimer] (cm^{-3}) at the condition of 278.15 K, $k_{\mathrm{coag}} = 2.0 \times 10^{-3}$ s^{-1}, [IA] = 1 × 10^7 cm^{-3} and [HIO$_2$] = 3.33 × 10^5 cm^{-3}.

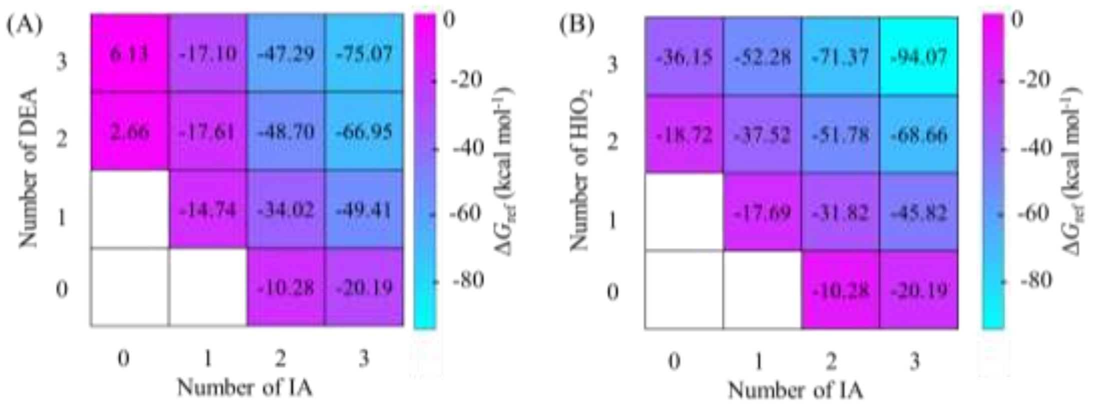

Figure S5. Formation free energy (ΔG) of $(IA)_x(DEA)_y$ (A) and $(IA)_x(HIO_2)_y$ (B) clusters (x = 0−3, y = 0−3) calculated at the DLPNO-CCSD(T)//M06-2X/aug-cc-pVTZ(-pp) level of theory. The calculations are performed at 278.15 K and 1 atm.

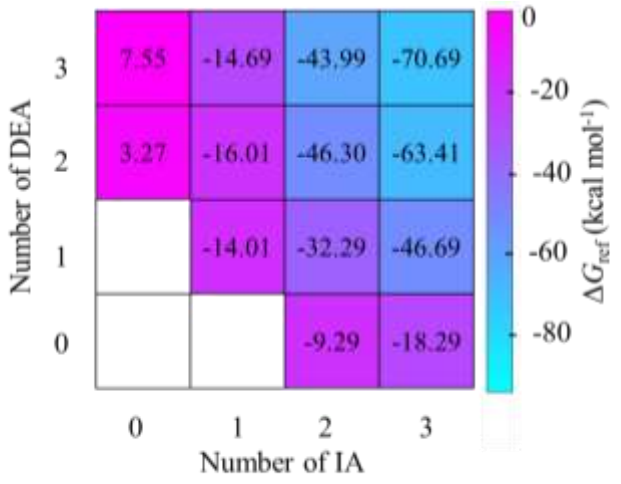

Figure S6. Formation free energy (ΔG) of $(IA)_x(DEA)_y$ clusters (x = 0−3, y = 0−3) calculated at the DLPNO-CCSD(T)//M06-2X/aug-cc-pVTZ(-pp) level of theory. The calculations are performed at 298.15 K and 1 atm.

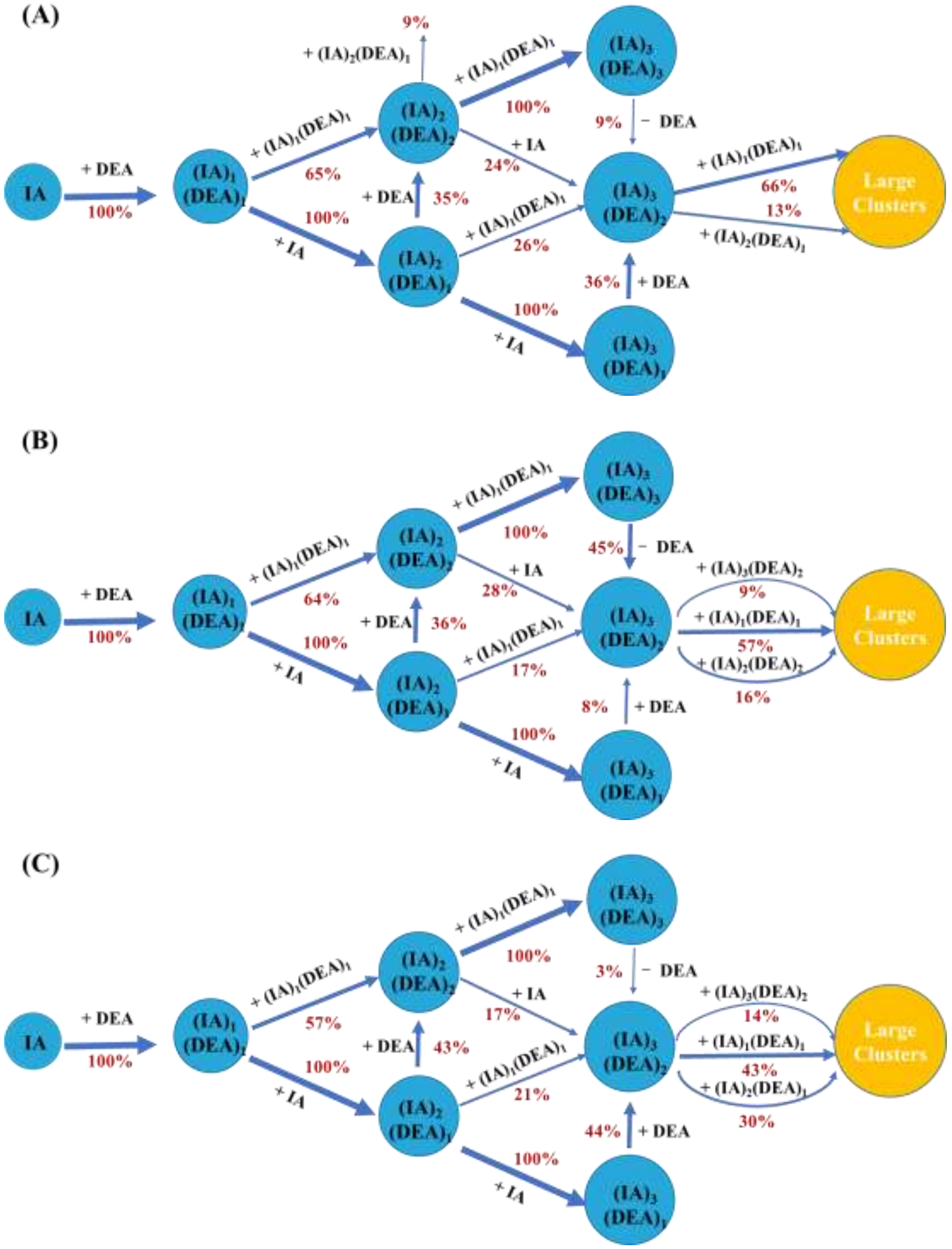

Figure S7. Cluster formation pathways for the IA-DEA system at [IA] = 10^7 cm^{-3}, [DEA] = 2.5×10^7 cm^{-3} and different simulation conditions. (A) 278.15 K and $k_{coag} = 2.0 \times 10^{-2}$ s^{-1}, (B) 263.15 K and $k_{coag} = 2.0 \times 10^{-3}$ s^{-1}, (C) 283.15 K and $k_{coag} = 2.0 \times 10^{-3}$ s^{-1}.

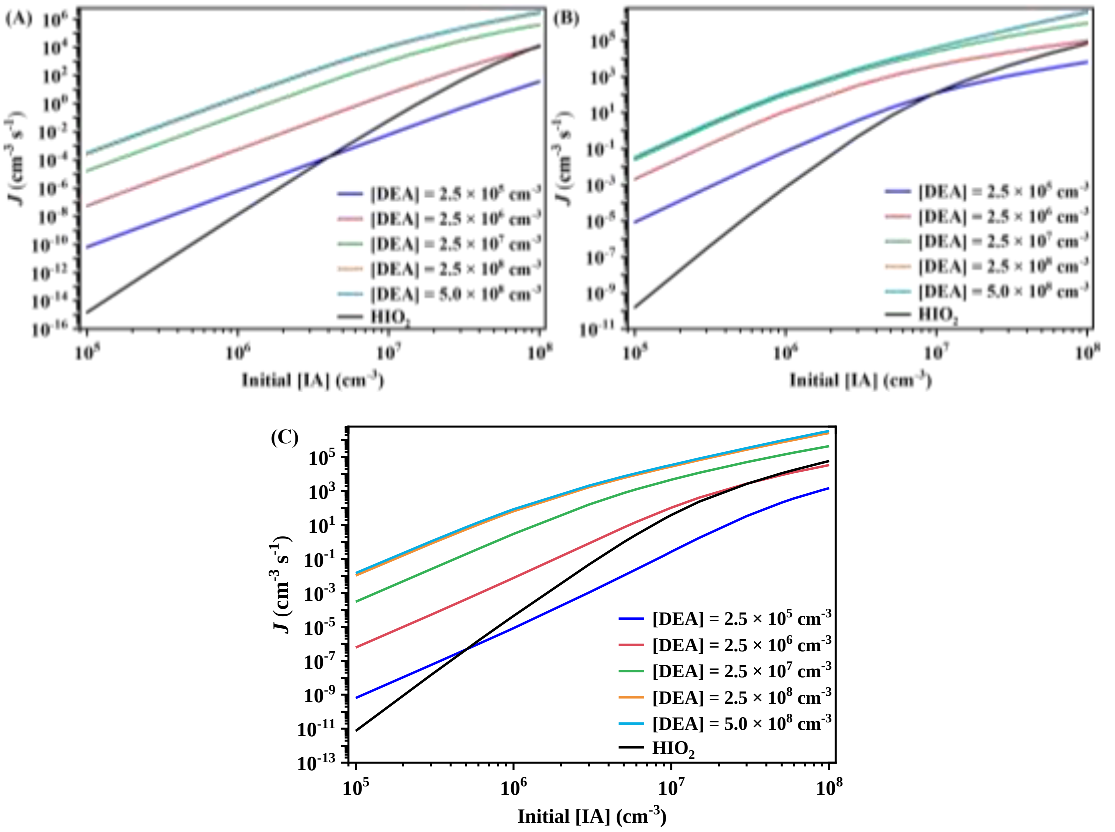

Figure S8. Simulated cluster formation rates J ($cm^{-3}s^{-1}$) of the systems as a function of [DEA] or [HIO_2] at 1 atm and different simulation conditions. (A) 278.15 K and $k_{coag} = 2.0 \times 10^{-2}\ s^{-1}$, (B) 263.15 K and $k_{coag} = 2.0 \times 10^{-3}\ s^{-1}$, (C) 283.15 K and $k_{coag} = 2.0 \times 10^{-3}\ s^{-1}$.

Coordinates of all optimized IA-DEA clusters

IA

I	-0.08737	-0.00013	-0.24652
O	1.773093	1.95E-05	0.233953
H	1.864735	0.000987	1.198838
O	-0.67165	1.427766	0.616698
O	-0.67149	-1.42847	0.616061

DEA

C	-2.44461	-0.36773	-0.02417
C	-1.21415	0.51925	0.017628
N	7.31E-08	-0.27304	-0.09279
C	1.214151	0.51925	0.017628
C	2.444608	-0.36773	-0.02417
H	-2.44354	-1.06851	0.812648
H	-2.46424	-0.94446	-0.94772
H	-3.35603	0.225209	0.038949
H	-1.23054	1.217236	-0.82289
H	-1.22472	1.130928	0.932828
H	1.15E-07	-0.99284	0.621907
H	1.224716	1.130928	0.932828
H	1.23054	1.217236	-0.82289
H	2.464244	-0.94446	-0.94772
H	2.443536	-1.06851	0.812648
H	3.356035	0.225209	0.038949

$(IA)_2$

I	1.621989	-0.17862	-0.26231
O	3.40271	0.037147	0.426673
H	3.405724	0.611803	1.204759
O	0.832866	-0.59952	1.288349
O	1.233649	1.561065	-0.4268
H	-0.32849	1.779658	0.09532
I	-1.74526	-0.15766	0.216808
O	-1.2871	1.659448	0.382773
O	-3.26775	-0.05005	-0.65476
O	-0.51843	-0.5943	-1.06052

$(IA)_3$

I	2.002237	-0.95199	-0.15717
O	3.216123	-1.51984	1.231178
H	4.101532	-1.17384	1.052774
O	-0.58421	1.888063	-0.13764

O	3.222924	-0.83171	-1.42293
I	0.138955	1.476651	1.491758
O	-1.42196	2.122538	2.428587
H	-2.16217	1.509152	2.330467
O	-0.25033	-0.24191	1.725728
O	1.928993	0.769883	0.507911
I	0.035274	1.382291	-2.22692
O	0.350805	0.841248	-4.04791
H	1.293011	0.67138	-4.19111
O	1.479126	2.378246	-2.0605
O	0.435123	-0.27957	-1.56636

$(DEA)_2$

C	1.440138	1.81954	-1.94191
C	2.102459	0.611527	-1.30556
N	1.550771	0.363015	0.018345
C	2.073616	-0.83965	0.654901
C	1.526622	-0.98188	2.063531
H	1.60552	2.713708	-1.33855
H	0.364473	1.659885	-2.02009
H	1.839858	2.008368	-2.93723
H	1.914173	-0.27751	-1.91202
H	3.193563	0.743092	-1.27807
H	1.729093	1.165011	0.614196
H	3.173063	-0.84759	0.668813
H	1.758213	-1.69265	0.048105
H	0.438797	-1.04336	2.040842
H	1.811402	-0.12428	2.676696
H	1.918905	-1.87819	2.541881
C	-1.74853	1.187131	2.117958
C	-2.35411	0.713387	0.808547
N	-1.56216	-0.36306	0.242598
C	-2.04957	-0.81973	-1.04552
C	-1.09201	-1.83818	-1.63752
H	-0.72694	1.537722	1.957905
H	-1.7126	0.368934	2.836672
H	-2.32497	2.005848	2.547183
H	-3.36799	0.3414	0.979536
H	-2.44752	1.564072	0.114103
H	-0.59681	-0.05249	0.140498
H	-2.1863	0.013925	-1.75366
H	-3.03594	-1.26958	-0.90341
H	-0.98048	-2.68752	-0.96398
H	-0.10696	-1.38826	-1.77874

H	-1.44153	-2.19898	-2.60409

$(DEA)_3$

N	2.50627	0.666711	-0.65152
C	1.995598	2.024011	-0.63239
C	3.651955	0.472048	0.21686
C	0.739827	2.125377	-1.48104
C	3.999793	-1.00347	0.300776
H	1.778384	2.365259	0.394107
H	2.767175	2.694781	-1.02074
H	4.497489	1.037366	-0.185
H	3.469289	0.864804	1.231194
H	1.760906	0.034681	-0.35818
H	0.966392	1.900957	-2.52307
H	-0.00448	1.402413	-1.13728
H	0.300869	3.122042	-1.42199
H	3.153575	-1.56458	0.703742
H	4.221695	-1.39638	-0.69099
H	4.859784	-1.17393	0.947337
N	0.15926	-1.26192	0.190593
C	0.249613	-1.87217	1.505233
C	0.144279	-2.22056	-0.90165
C	0.399941	-0.80302	2.572741
C	-0.1058	-1.51138	-2.22054
H	-0.62226	-2.50868	1.723511
H	1.121204	-2.53244	1.514039
H	1.116549	-2.72156	-0.92679
H	-0.60844	-3.00984	-0.7466
H	-0.66988	-0.66908	0.149531
H	1.294572	-0.20792	2.381978
H	-0.45737	-0.12792	2.558969
H	0.474369	-1.24072	3.567564
H	-1.05766	-0.97482	-2.18895
H	0.685287	-0.78679	-2.41375
H	-0.14344	-2.22006	-3.04676
N	-2.52942	0.523489	0.345937
C	-3.50291	-0.0415	-0.57921
C	-2.44434	1.977555	0.282924
C	-3.59787	-1.54567	-0.4023
C	-1.42777	2.495993	1.282068
H	-4.49476	0.416875	-0.45825
H	-3.16998	0.196693	-1.59263
H	-2.13923	2.245212	-0.73092
H	-3.42239	2.449401	0.453983

H	-2.77026	0.24507	1.292625
H	-2.62509	-2.0112	-0.56224
H	-3.92999	-1.79541	0.607086
H	-4.30854	-1.9784	-1.10469
H	-1.7325	2.259871	2.30365
H	-0.45527	2.036331	1.103568
H	-1.32096	3.577321	1.20616

$(IA)_1(DEA)_1$

O	2.016037	1.275634	-0.02584
I	1.387869	-0.3912	-0.03846
O	0.245631	-0.46509	1.375488
H	-1.2708	0.031093	0.802028
O	0.204076	-0.42867	-1.41901
C	-1.11216	2.556562	0.038506
C	-2.34371	1.671832	0.048208
N	-1.94837	0.244818	0.021468
C	-3.06394	-0.72528	0.026931
C	-2.52204	-2.14167	-0.0022
H	-0.50948	2.380113	-0.85174
H	-0.47816	2.355862	0.901426
H	-1.41064	3.602819	0.05827
H	-2.94582	1.826886	0.943502
H	-2.97736	1.851398	-0.82035
H	-1.29814	0.053431	-0.7869
H	-3.68795	-0.51555	-0.84158
H	-3.65761	-0.54027	0.921925
H	-1.89102	-2.32597	0.866515
H	-1.92113	-2.3013	-0.89677
H	-3.34419	-2.85433	0.001869

$(IA)_1(DEA)_2$

O	-0.60377	-0.45092	1.410659
I	-1.55996	-0.30148	-0.10445
O	-1.09586	1.363306	-0.66377
H	0.467471	1.471146	-0.29165
O	-0.68673	-1.38206	-1.24174
N	1.5052	1.461691	-0.06234
C	1.679545	1.892385	1.345484
C	2.207696	2.249944	-1.09915
H	1.81169	0.44087	-0.12793
H	1.063299	1.20852	1.927761
H	3.280746	2.096632	-0.98564
N	2.127641	-1.28467	-0.10487

C	2.319826	-1.76103	1.268438
C	3.01048	-1.90801	-1.09032
H	1.162877	-1.48171	-0.38314
H	1.7506	-1.10183	1.925035
H	3.379398	-1.65178	1.515751
H	4.044133	-1.72376	-0.78637
H	2.875531	-2.99436	-1.12312
C	2.746809	-1.3254	-2.46798
H	3.330414	-1.84128	-3.22926
H	1.688903	-1.41761	-2.71759
H	3.013332	-0.26814	-2.49532
C	1.861181	-3.19446	1.503246
H	2.426664	-3.90787	0.904624
H	1.980807	-3.46729	2.551558
H	0.804854	-3.2837	1.247054
H	1.907111	1.81687	-2.05213
C	1.839802	3.720106	-1.04678
H	0.75601	3.838749	-1.06441
H	2.23036	4.204819	-0.15285
H	2.25464	4.232751	-1.9124
H	1.260364	2.891649	1.452111
C	3.13341	1.843503	1.772602
H	3.737036	2.589089	1.254883
H	3.20989	2.036626	2.840811
H	3.555581	0.857114	1.574877

$(IA)_1(DEA)_3$

O	-0.22368	-0.67151	1.086843
I	-0.18693	-0.03959	-0.58236
O	1.114563	1.331564	-0.34616
H	2.030146	0.914936	-0.11075
O	0.846611	-1.18405	-1.49099
C	4.220666	0.705257	-2.02179
C	3.95873	-0.38517	-1.00018
N	3.429815	0.189753	0.240473
C	3.144427	-0.81835	1.26731
C	2.690539	-0.15703	2.554253
H	4.951085	1.427133	-1.65083
H	3.296575	1.236715	-2.24768
H	4.609296	0.279727	-2.94562
H	3.200526	-1.07351	-1.37424
H	4.872009	-0.95857	-0.80065
H	4.091097	0.866848	0.609465
H	4.025063	-1.44826	1.442339

H	2.351139	-1.4584	0.876919
H	1.786823	0.42445	2.376873
H	3.467868	0.498167	2.953361
H	2.462592	-0.90943	3.307406
C	-0.80911	-3.74623	-0.18275
C	-1.48754	-3.11919	-1.38507
N	-2.08423	-1.82325	-1.03942
C	-2.92569	-1.27176	-2.09884
C	-3.76472	-0.11505	-1.58571
H	-1.52303	-3.90636	0.627722
H	-0.01352	-3.10216	0.183929
H	-0.38695	-4.71213	-0.45598
H	-0.75577	-2.93761	-2.17223
H	-2.2615	-3.78735	-1.78264
H	-2.61728	-1.91582	-0.17933
H	-3.57359	-2.04709	-2.52395
H	-2.26396	-0.94002	-2.905
H	-3.13639	0.671364	-1.16581
H	-4.44451	-0.45244	-0.80143
H	-4.36142	0.312811	-2.38944
C	-0.81097	3.963017	-0.58854
C	-0.93853	3.370789	0.801767
N	-1.80225	2.19161	0.785971
C	-2.06166	1.663876	2.123964
C	-3.1065	0.566021	2.076183
H	-1.78837	4.254156	-0.97949
H	-0.36046	3.24015	-1.26701
H	-0.18023	4.850742	-0.57338
H	0.041643	3.054356	1.159461
H	-1.32036	4.126701	1.502073
H	-2.68889	2.449196	0.361734
H	-2.38357	2.463623	2.805396
H	-1.12345	1.256979	2.503565
H	-2.73551	-0.26693	1.48018
H	-4.03976	0.931508	1.640861
H	-3.32417	0.196151	3.077035

$(IA)_2(DEA)_1$

O	-1.22838	-1.82072	1.051226
I	-2.04668	-1.17709	-0.41931
O	-2.79756	0.364394	0.131022
H	-1.57127	1.526858	0.404003
O	-0.61749	-0.61331	-1.36616
C	-0.41084	1.199641	2.753447

C	-0.55446	2.478514	1.95266
N	-0.74512	2.156171	0.517917
C	-0.88695	3.328098	-0.37663
C	-1.10577	2.861598	-1.80215
H	0.462105	0.637143	2.422685
H	-1.29057	0.56691	2.637871
H	-0.28765	1.435398	3.808587
H	-1.4139	3.065924	2.274839
H	0.338196	3.099069	2.025448
H	0.099021	1.617467	0.196153
H	0.026167	3.915085	-0.28276
H	-1.72234	3.925339	-0.0123
H	-2.0198	2.27347	-1.87554
H	-0.27388	2.238085	-2.12737
H	-1.18528	3.719329	-2.46689
I	2.034153	-0.5664	-0.5669
O	1.361762	-1.44818	0.959954
H	0.362024	-1.58702	0.965994
O	3.7508	-0.71557	-0.18729
O	1.628286	1.135938	-0.16833

$(IA)_2(DEA)_2$

O	-3.41042	-0.00741	0.597442
I	-3.11681	-0.01115	-1.16395
O	-2.08888	1.44919	-1.44292
H	-0.71494	1.808751	-0.57359
O	-1.95248	-1.37244	-1.40645
N	0.08409	2.346267	-0.15853
C	-0.38839	3.006028	1.083016
C	0.567951	3.282787	-1.2006
H	0.884624	1.715876	0.079527
H	1.383425	3.858146	-0.76364
N	-0.00548	-2.44029	0.102212
C	-0.60352	-2.94364	1.36274
C	0.369012	-3.5022	-0.86173
H	-0.70233	-1.82508	-0.37825
H	0.121349	-3.62395	1.809682
H	1.078467	-4.15827	-0.35843
H	-0.53585	-4.06451	-1.08978
C	0.968748	-2.88153	-2.10747
H	1.246721	-3.66107	-2.81428
H	0.243905	-2.22149	-2.58259
H	1.860128	-2.31048	-1.84994
H	-1.18108	3.696419	0.794899

I	3.039053	-0.06001	1.473083
O	2.46353	-1.58645	0.694744
H	0.870508	-1.91398	0.337336
O	1.972081	0.135036	2.891619
O	2.470076	1.214314	0.324192
H	-1.49493	-3.50915	1.09105
C	-0.93138	-1.78677	2.284284
H	-1.66551	-1.12517	1.822005
H	-1.35853	-2.16742	3.210684
H	-0.03046	-1.22318	2.53227
H	0.449472	3.579192	1.4801
H	-0.25593	3.955574	-1.43646
C	-0.87844	1.968631	2.072337
H	-0.06743	1.295648	2.354689
H	-1.23379	2.463599	2.974722
H	-1.70568	1.394414	1.651927
C	1.023387	2.507977	-2.42085
H	0.195591	1.927629	-2.82696
H	1.375876	3.19541	-3.18748
H	1.837433	1.834912	-2.15418

$(IA)_2(DEA)_3$

N	-1.78189	2.079398	0.319564
C	-2.24286	2.973638	-0.77275
C	-2.19089	2.509414	1.67959
C	-2.06471	2.300038	-2.11827
C	-1.60228	1.569255	2.712566
H	-3.29031	3.201035	-0.5766
H	-1.6586	3.890992	-0.7028
H	-1.83603	3.530414	1.815809
H	-3.27998	2.501275	1.697863
H	-2.20907	1.142848	0.16072
H	-1.00914	2.099994	-2.30076
H	-2.64065	1.374981	-2.15448
H	-2.42041	2.961155	-2.9066
H	-1.88949	0.53791	2.501135
H	-0.5153	1.636218	2.703045
H	-1.96201	1.835374	3.704879
N	2.7171	-1.39796	0.47647
C	3.837483	-2.16381	-0.06954
C	2.604662	-1.42368	1.936014
C	5.214596	-1.62171	0.29615
C	1.397419	-0.61021	2.374959
H	3.735179	-3.20014	0.263821

H	3.731301	-2.16686	-1.15705
H	2.504504	-2.46671	2.249531
H	3.506267	-1.02901	2.416015
H	2.813826	-0.42271	0.177242
H	5.284859	-0.57095	0.012296
H	5.408685	-1.70375	1.364716
H	5.994636	-2.1743	-0.22717
H	1.464243	0.405591	1.979763
H	0.462502	-1.0519	2.023741
H	1.346486	-0.55108	3.461904
N	0.269389	-1.81037	-0.73373
C	0.078976	-0.52745	-1.46188
C	0.204582	-3.03457	-1.56405
C	1.045236	-0.36447	-2.61738
C	0.186035	-4.24949	-0.65775
H	-0.96034	-0.49615	-1.79135
H	0.245985	0.259183	-0.72167
H	-0.70135	-2.97651	-2.16498
H	1.075536	-3.03953	-2.21722
H	1.219611	-1.75418	-0.23647
H	2.074044	-0.50005	-2.28039
H	0.826946	-1.06043	-3.42606
H	0.971019	0.653975	-2.99459
H	1.070951	-4.27237	-0.01951
H	-0.69853	-4.23031	-0.02151
H	0.16708	-5.16194	-1.25011
H	-0.4643	-1.86618	0.001815
O	-3.56061	0.080533	0.018115
O	-1.7934	-1.77752	1.161509
O	-2.83289	-2.28268	-1.39031
H	-0.73563	2.053844	0.320937
O	3.016407	1.31505	-0.54218
O	0.828914	2.522081	0.786982
O	1.12754	2.913416	-1.96927
I	2.097769	2.857516	-0.46539
I	-3.33838	-1.69887	0.224004

$(IA)_3(DEA)_1$

O	2.172119	1.719053	1.352202
I	3.042284	0.227672	0.870411
O	2.668679	-0.99581	2.127515
H	1.614507	-2.16761	1.448185
O	2.068989	-0.40447	-0.54114
O	-3.57595	-1.03987	0.070372

I	-3.15139	0.173295	-1.18003
O	-2.77849	1.688975	-0.29592
H	-1.23518	1.893168	0.568576
O	-1.51595	-0.43011	-1.72422
C	-1.20935	1.657745	3.197725
C	-0.88197	2.870951	2.350792
N	-0.46484	2.439349	0.99612
C	-0.09772	3.535165	0.071152
C	0.298168	2.955394	-1.27214
H	-0.33817	1.010146	3.290364
H	-2.01208	1.07827	2.742611
H	-1.52178	1.974869	4.190727
H	-1.74441	3.525412	2.227895
H	-0.05785	3.447081	2.770309
H	0.3538	1.816685	1.092125
H	0.725281	4.080043	0.531551
H	-0.96209	4.192873	-0.01241
H	-0.53431	2.406844	-1.71063
H	1.13792	2.270568	-1.15754
H	0.588534	3.752994	-1.95318
I	-0.0072	-1.58057	-0.23912
O	-1.47337	-2.73159	0.075912
H	-2.30609	-2.19639	0.159293
O	0.99928	-2.71348	0.890284
O	-0.39554	-0.28926	0.90506

$(IA)_3(DEA)_2$

I	0.736316	-1.82226	-1.17128
O	-0.97663	-2.41435	-1.74682
H	-1.68803	-2.09264	-1.11929
O	1.060075	-2.9586	0.148901
O	0.161143	-0.33106	-0.35163
O	-2.78947	-1.49857	-0.06827
I	-3.31841	0.225026	-0.0645
O	-2.50598	0.899741	1.399966
H	-1.08679	0.046259	1.904205
O	-2.45716	1.026932	-1.42347
N	-0.27985	-0.37485	2.41202
C	-0.57598	-1.80437	2.693092
C	0.02963	0.474259	3.585724
H	0.529287	-0.33272	1.775495
H	0.943071	0.081995	4.030067
N	0.134599	2.103398	-1.48587
C	0.708383	1.640998	-2.77776

C	-0.50268	3.439031	-1.50354
H	-0.57177	1.411833	-1.18377
H	1.466324	0.900858	-2.52157
H	0.213701	4.141256	-1.92856
H	-1.37321	3.374017	-2.15312
C	-0.89471	3.824801	-0.09129
H	-1.39574	4.790923	-0.09151
H	-1.56818	3.079336	0.333056
H	-0.01033	3.894782	0.54318
H	0.296707	-2.22071	3.194971
I	3.33212	0.444414	0.392692
O	2.485888	1.961414	-0.08613
H	0.909071	2.069857	-0.78999
O	2.900768	-0.735	-0.92467
O	2.455813	-0.1205	1.846862
H	1.221095	2.4919	-3.22513
C	-0.34854	1.062558	-3.69746
H	-1.12463	1.790945	-3.928
H	0.116242	0.749494	-4.63089
H	-0.83748	0.205033	-3.23484
H	-0.66003	-2.28811	1.723172
H	-0.78857	0.377505	4.295849
C	-1.85031	-1.97477	3.495196
H	-2.67334	-1.4511	3.008816
H	-2.10245	-3.03242	3.538684
H	-1.75504	-1.61229	4.517508
C	0.195208	1.913857	3.137912
H	-0.7352	2.281496	2.705235
H	0.457506	2.540873	3.98828
H	0.987966	1.988415	2.393151

$(IA)_3(DEA)_3$

N	-0.37916	2.072771	-0.48204
C	-0.82266	2.538473	-1.82469
C	-0.29963	3.158188	0.533822
C	-0.82232	1.387185	-2.80839
C	1.023493	3.895778	0.47733
H	-1.8194	2.952646	-1.68575
H	-0.13056	3.319013	-2.13494
H	-1.15325	3.810454	0.35625
H	-0.43736	2.682603	1.504196
H	-1.03474	1.353659	-0.12946
H	0.197879	1.054418	-2.99704
H	-1.40797	0.550074	-2.42739

H	-1.26472	1.709592	-3.74931
H	1.848013	3.229521	0.730724
H	1.213198	4.302538	-0.51471
H	1.005741	4.719611	1.18903
N	-2.79129	-1.76604	-0.4862
C	-4.05155	-1.73863	-1.27031
C	-2.7968	-2.75567	0.615339
C	-3.98653	-0.65864	-2.32967
C	-1.46652	-2.75508	1.344697
H	-4.16234	-2.72711	-1.71567
H	-4.86658	-1.55177	-0.57517
H	-3.61885	-2.48417	1.278151
H	-3.01117	-3.72957	0.176462
H	-1.99054	-1.96735	-1.15694
H	-3.86103	0.321765	-1.8662
H	-3.16541	-0.84713	-3.02086
H	-4.91907	-0.63998	-2.89055
H	-0.65724	-3.08006	0.689305
H	-1.24557	-1.74843	1.702132
H	-1.51898	-3.43261	2.196205
N	2.180012	-1.1514	1.560319
C	2.989991	-2.39474	1.492264
C	1.495329	-0.88295	2.844962
C	4.15594	-2.3575	2.46069
C	0.689508	0.396803	2.726469
H	2.317996	-3.23173	1.675384
H	3.325616	-2.47048	0.461526
H	0.861785	-1.74275	3.064827
H	2.251903	-0.80338	3.622851
H	2.777654	-0.32834	1.323767
H	4.746499	-1.45156	2.314832
H	3.838715	-2.40191	3.501482
H	4.802058	-3.21344	2.276102
H	1.347653	1.224337	2.454371
H	-0.08844	0.296455	1.968273
H	0.211687	0.628955	3.676978
H	-2.60838	-0.8387	-0.06041
O	-3.52442	2.731346	-0.1367
O	-2.24327	0.569825	1.0657
O	-5.00884	0.402499	0.670622
H	1.461288	-1.13871	0.795536
O	1.355578	-3.20307	-0.91511
O	0.394487	-0.63634	-0.45903
O	-0.91752	-2.24747	-2.30238

H	0.535383	1.609683	-0.60172
O	3.488298	1.13337	0.725164
O	2.163453	1.801855	-1.69132
O	3.350331	-0.75983	-1.34865
I	-3.76401	1.568976	1.206625
I	3.645491	0.995109	-1.0721
I	0.769681	-1.78225	-1.826

References

(1) Sipila, M.; Sarnela, N.; Jokinen, T.; Henschel, H.; Junninen, H.; Kontkanen, J.; Richters, S.; Kangasluoma, J.; Franchin, A.; Perakyla, O.; Rissanen, M. P.; Ehn, M.; Vehkamaki, H.; Kurten, T.; Berndt, T.; Petaja, T.; Worsnop, D.; Ceburnis, D.; Kerminen, V. M.; Kulmala, M.; O'Dowd, C. Molecular-Scale Evidence of Aerosol Particle Formation Via Sequential Addition of HIO_3. *Nature* **2016**, 537, 532-534.

(2) He, X. C.; Tham, Y. J.; Dada, L.; Wang, M.; Finkenzeller, H.; Stolzenburg, D.; Iyer, S.; Simon, M.; Kürten, A.; Shen, J.; Rörup, B.; Rissanen, M.; Schobesberger, S.; Baalbaki, R.; Wang, D. S.; Koenig, T. K.; Jokinen, T.; Sarnela, N.; Beck, L. J.; Almeida, J.; Amanatidis, S.; Amorim, A.; Ataei, F.; Baccarini, A.; Bertozzi, B.; Bianchi, F.; Brilke, S.; Caudillo, L.; Chen, D.; Chiu, R.; Chu, B.; Dias, A.; Ding, A.; Dommen, J.; Duplissy, J.; El Haddad, I.; Gonzalez Carracedo, L.; Granzin, M.; Hansel, A.; Heinritzi, M.; Hofbauer, V.; Junninen, H.; Kangasluoma, J.; Kemppainen, D.; Kim, C.; Kong, W.; Krechmer, J. E.; Kvashin, A.; Laitinen, T.; Lamkaddam, H.; Lee, C. P.; Lehtipalo, K.; Leiminger, M.; Li, Z.; Makhmutov, V.; Manninen, H. E.; Marie, G.; Marten, R.; Mathot, S.; Mauldin, R. L.; Mentler, B.; Möhler, O.; Müller, T.; Nie, W.; Onnela, A.; Petäjä, T.; Pfeifer, J.; Philippov, M.; Ranjithkumar, A.; Saiz-Lopez, A.; Salma, I.; Scholz, W.; Schuchmann, S.; Schulze, B.; Steiner, G.; Stozhkov, Y.; Tauber, C.; Tomé, A.; Thakur, R. C.; Väisänen, O.; Vazquez-Pufleau, M.; Wagner, A. C.; Wang, Y.; Weber, S. K.; Winkler, P. M.; Wu, Y.; Xiao, M.; Yan, C.; Ye, Q.; Ylisirniö, A.; Zauner-Wieczorek, M.; Zha, Q.; Zhou, P.; Flagan, R. C.; Curtius, J.; Baltensperger, U.; Kulmala, M.; Kerminen, V.-M.; Kurtén, T.; Donahue, N. M.; Volkamer, R.; Kirkby, J.; Worsnop, D. R.; Sipilä, M. Role of Iodine Oxoacids in Atmospheric Aerosol Nucleation. *Science* **2021**, 371, 589-595.

(3) Huang, R. J.; Hoffmann, T.; Ovadnevaite, J.; Laaksonen, A.; Kokkola, H.; Xu, W.; Xu, W.; Ceburnis, D.; Zhang, R.; Seinfeld, J. H.; O'Dowd, C. Heterogeneous Iodine-Organic Chemistry Fast-Tracks Marine New Particle Formation. *Proc Natl Acad Sci U S A* **2022**, 119, e2201729119.

(4) Berresheim, H.; Elste, T.; Tremmel, H. G.; Allen, A. G.; Hansson, H. C.; Rosman, K.; Dal Maso, M.; Makela, J. M.; Kulmala, M.; O'Dowd, C. D. Gas-Aerosol Relationships of H_2SO_4, MSA, and OH: Observations in the Coastal Marine Boundary Layer at Mace Head, Ireland. *J. Geophys. Res.-Atmos.* **2002**, 107, 12.

(5) Beck, L. J.; Sarnela, N.; Junninen, H.; Hoppe, C. J. M.; Garmash, O.; Bianchi, F.; Riva, M.; Rose, C.; Peräkylä, O.; Wimmer, D.; Kausiala, O.; Jokinen, T.; Ahonen, L.; Mikkilä, J.; Hakala, J.; He, X. C.; Kontkanen, J.; Wolf, K. K. E.; Cappelletti, D.; Mazzola, M.; Traversi, R.; Petroselli, C.; Viola, A. P.; Vitale, V.; Lange, R.; Massling, A.; Nøjgaard, J. K.; Krejci, R.; Karlsson, L.; Zieger, P.; Jang, S.; Lee, K.; Vakkari, V.; Lampilahti, J.; Thakur, R. C.; Leino, K.; Kangasluoma, J.; Duplissy, E. M.; Siivola, E.; Marbouti, M.; Tham, Y. J.; Saiz-Lopez, A.; Petäjä, T.; Ehn, M.; Worsnop, D. R.; Skov, H.; Kulmala, M.; Kerminen, V. M.; Sipilä, M. Differing Mechanisms of New Particle Formation at Two Arctic Sites. *Geophys. Res. Lett.* **2021**, 48, e2020GL091334.

(6) Chen, H.; Finlayson-Pitts, B. J. New Particle Formation from Methanesulfonic Acid and Amines/Ammonia as a Function of Temperature. *Environ. Sci. Technol.* **2017**, 51,

243-252.
(7) Ning, A.; Zhang, H.; Zhang, X.; Li, Z.; Zhang, Y.; Xu, Y.; Ge, M. A Molecular-Scale Study on the Role of Methanesulfinic Acid in Marine New Particle Formation. *Atmos. Environ.* **2020**, 227, 117378.
(8) Liu, J.; Gunsch, M. J.; Moffett, C. E.; Xu, L.; El Asmar, R.; Zhang, Q.; Watson, T. B.; Allen, H. M.; Crounse, J. D.; St. Clair, J.; Kim, M.; Wennberg, P. O.; Weber, R. J.; Sheesley, R. J.; Pratt, K. A. Hydroxymethanesulfonate (HMS) Formation During Summertime Fog in an Arctic Oil Field. *Environ. Sci. Technol. Lett.* **2021**, 8, 511-518.
(9) Wespes, C.; Hurtmans, D.; Herbin, H.; Barret, B.; Turquety, S.; Hadji-Lazaro, J.; Clerbaux, C.; Coheur, P. F. First Global Distributions of Nitric Acid in the Troposphere and the Stratosphere Derived from Infrared Satellite Measurements. *J. Geophys. Res.-Atmos.* **2007**, 112, D13311.
(10) Edtbauer, A.; Stönner, C.; Pfannerstill, E. Y.; Berasategui, M.; Walter, D.; Crowley, J. N.; Lelieveld, J.; Williams, J. A New Marine Biogenic Emission: Methane Sulfonamide (Msam), Dimethyl Sulfide (DMS), and Dimethyl Sulfone ($DMSO_2$) Measured in Air over the Arabian Sea. *Atmos. Chem. Phys.* **2020**, 20, 6081-6094.
(11) Xia, D. M.; Chen, J.; Yu, H.; Xie, H. B.; Wang, Y.; Wang, Z.; Xu, T.; Allen, D. T. Formation Mechanisms of Iodine–Ammonia Clusters in Polluted Coastal Areas Unveiled by Thermodynamics and Kinetic Simulations. *Environ. Sci. Technol.* **2020**, 54, 9235–9242.
(12) Chen, D.; Yao, X.; Chan, C. K.; Tian, X.; Chu, Y.; Clegg, S. L.; Shen, Y.; Gao, Y.; Gao, H. Competitive Uptake of Dimethylamine and Trimethylamine against Ammonia on Acidic Particles in Marine Atmospheres. *Environ. Sci. Technol.* **2022**, 56, 5430-5439.
(13) van Pinxteren, M.; Fomba, K. W.; van Pinxteren, D.; Triesch, N.; Hoffmann, E. H.; Cree, C. H. L.; Fitzsimons, M. F.; von Tümpling, W.; Herrmann, H. Aliphatic Amines at the Cape Verde Atmospheric Observatory: Abundance, Origins and Sea-Air Fluxes. *Atmos. Environ.* **2019**, 203, 183-195.
(14) Liu, Z.; Li, M.; Wang, X.; Liang, Y.; Jiang, Y.; Chen, J.; Mu, J.; Zhu, Y.; Meng, H.; Yang, L.; Hou, K.; Wang, Y.; Xue, L. Large Contributions of Anthropogenic Sources to Amines in Fine Particles at a Coastal Area in Northern China in Winter. *Sci. Total Environ.* **2022**, 839, 156281.
(15) Hu, Q. J.; Yu, P. R.; Zhu, Y. J.; Li, K.; Gao, H. W.; Yao, X. H. Concentration, Size Distribution, and Formation of Trimethylaminium and Dimethylaminium Ions in Atmospheric Particles over Marginal Seas of China. *J. Atmos. Sci.* **2015**, 72, 3487-3498.
(16) van Pinxteren, M.; Fiedler, B.; van Pinxteren, D.; Iinuma, Y.; Körtzinger, A.; Herrmann, H. Chemical Characterization of Sub-Micrometer Aerosol Particles in the Tropical Atlantic Ocean: Marine and Biomass Burning Influences. *J. Atmos. Chem.* **2015**, 72, 105-125.
(17) Tzitzikalaki, E.; Kalivitis, N.; Kanakidou, M. Observations of Gas-Phase Alkylamines at a Coastal Site in the East Mediterranean Atmosphere. *Atmosphere* **2021**, 12, 1454.
(18) Chen, D.; Shen, Y.; Wang, J.; Gao, Y.; Gao, H.; Yao, X. Mapping Gaseous Dimethylamine, Trimethylamine, Ammonia, and Their Particulate Counterparts in Marine Atmospheres of China's Marginal Seas – Part 1: Differentiating Marine

Emission from Continental Transport. *Atmos. Chem. Phys.* **2021**, 21, 16413-16425.
(19) Ho, S. S. H.; Li, L.; Qu, L.; Cao, J.; Lui, K. H.; Niu, X.; Lee, S. C.; Ho, K. F. Seasonal Behavior of Water-Soluble Organic Nitrogen in Fine Particulate Matter ($PM_{2.5}$) at Urban Coastal Environments in Hong Kong. *Air Qual. Atmos. Hlth.* **2018**, 12, 389-399.
(20) Feltracco, M.; Barbaro, E.; Kirchgeorg, T.; Spolaor, A.; Turetta, C.; Zangrando, R.; Barbante, C.; Gambaro, A. Free and Combined L- and D-Amino Acids in Arctic Aerosol. *Chemosphere* **2019**, 220, 412-421.
(21) Zhang, Q.; Anastasio, C. Free and Combined Amino Compounds in Atmospheric Fine Particles ($PM_{2.5}$) and Fog Waters from Northern California. *Atmos. Environ.* **2003**, 37, 2247-2258.
(22) Mandalakis, M.; Apostolaki, M.; Tziaras, T.; Polymenakou, P.; Stephanou, E. G. Free and Combined Amino Acids in Marine Background Atmospheric Aerosols over the Eastern Mediterranean. *Atmos. Environ.* **2011**, 45, 1003-1009.
(23) Scalabrin, E.; Zangrando, R.; Barbaro, E.; Kehrwald, N. M.; Gabrieli, J.; Barbante, C.; Gambaro, A. Amino Acids in Arctic Aerosols. *Atmos. Chem. Phys.* **2012**, 12, 10453-10463.
(24) Yu, Z.; Li, Y. Marine Volatile Organic Compounds and Their Impacts on Marine Aerosol-a Review. *Sci. Total Environ.* **2021**, 768, 145054.
(25) Schlundt, C.; Tegtmeier, S.; Lennartz, S. T.; Bracher, A.; Cheah, W.; Krüger, K.; Quack, B.; Marandino, C. A. Oxygenated Volatile Organic Carbon in the Western Pacific Convective Center: Ocean Cycling, Air–Sea Gas Exchange and Atmospheric Transport. *Atmos. Chem. Phys.* **2017**, 17, 10837-10854.
(26) Vichi, F.; Imperiali, A.; Frattoni, M.; Perilli, M.; Benedetti, P.; Esposito, G.; Cecinato, A. Air Pollution Survey across the Western Mediterranean Sea: Overview on Oxygenated Volatile Hydrocarbons (OVOCs) and Other Gaseous Pollutants. *Environ Sci Pollut Res Int.* **2019**, 26, 16781-16799.
(27) van Pinxteren, M.; Herrmann, H. Glyoxal and Methylglyoxal in Atlantic Seawater and Marine Aerosol Particles: Method Development and First Application During the Polarstern Cruise ANT XXVII/4. *Atmos. Chem. Phys.* **2013**, 13, 11791-11802.
(28) Zhu, Y.; Tilgner, A.; Hans Hoffmann, E.; Herrmann, H.; Kawamura, K.; Xue, L.; Yang, L.; Wang, W. Molecular Distributions of Dicarboxylic Acids, Oxocarboxylic Acids, and A-Dicarbonyls in Aerosols over Tuoji Island in the Bohai Sea: Effects of East Asian Continental Outflow. *Atmos. Res.* **2022**, 272, 106154.
(29) Khwaja, H. A. Atmospheric Concentrations of Carboxylic-Acids and Related-Compounds at a Semiurban Site. *Atmos. Environ.* **1995**, 29, 127-139.
(30) Mungall, E. L.; Abbatt, J. P. D.; Wentzell, J. J. B.; Wentworth, G. R.; Murphy, J. G.; Kunkel, D.; Gute, E.; Tarasick, D. W.; Sharma, S.; Cox, C. J.; Uttal, T.; Liggio, J. High Gas-Phase Mixing Ratios of Formic and Acetic Acid in the High Arctic. *Atmos. Chem. Phys.* **2018**, 18, 10237-10254.
(31) Hansen, A. M. K.; Kristensen, K.; Nguyen, Q. T.; Zare, A.; Cozzi, F.; Nøjgaard, J. K.; Skov, H.; Brandt, J.; Christensen, J. H.; Ström, J.; Tunved, P.; Krejci, R.; Glasius, M. Organosulfates and Organic Acids in Arctic Aerosols: Speciation, Annual Variation and Concentration Levels. *Atmos. Chem. Phys.* **2014**, 14, 7807-7823.

(32) Fu, P.; Kawamura, K.; Usukura, K.; Miura, K. Dicarboxylic Acids, Ketocarboxylic Acids and Glyoxal in the Marine Aerosols Collected During a Round-the-World Cruise. *Mar. Chem.* **2013**, 148, 22-32.
(33) Baboukas, E. D.; Kanakidou, M.; Mihalopoulos, N. Carboxylic Acids in Gas and Particulate Phase above the Atlantic Ocean. *J. Geophys. Res.-Atmos.* **2000**, 105, 14459-14471.
(34) Hoque, M. M. M.; Kawamura, K.; Nagayama, T.; Kunwar, B.; Peltzer, E. T.; Gagosian, R. B. Molecular Characteristics of Water-Soluble Dicarboxylic Acids, Ω-Oxocarboxylic Acids, Pyruvic Acid and α-Dicarbonyls in the Aerosols from the Eastern North Pacific. *Mar. Chem.* **2020**, 224, 103812.
(35) Fang, X.; Wang, Q.; Zhao, Z.; Tang, J.; Tian, C.; Yao, Y.; Yu, J.; Sun, H. Distribution and Dry Deposition of Alternative and Legacy Perfluoroalkyl and Polyfluoroalkyl Substances in the Air above the Bohai and Yellow Seas, China. *Atmos. Environ.* **2018**, 192, 128-135.
(36) Yamazaki, E.; Taniyasu, S.; Wang, X.; Yamashita, N. Per- and Polyfluoroalkyl Substances in Surface Water, Gas and Particle in Open Ocean and Coastal Environment. *Chemosphere* **2021**, 272, 129869.
(37) Yang, J.; Zhao, W.; Wei, L.; Zhang, Q.; Zhao, Y.; Hu, W.; Wu, L.; Li, X.; Pavuluri, C. M.; Pan, X.; Sun, Y.; Wang, Z.; Liu, C.-Q.; Kawamura, K.; Fu, P. Molecular and Spatial Distributions of Dicarboxylic Acids, Oxocarboxylic Acids, and α-Dicarbonyls in Marine Aerosols from the South China Sea to the Eastern Indian Ocean. *Atmos. Chem. Phys.* **2020**, 20, 6841-6860.
(38) Deshmukh, D. K.; Mozammel Haque, M.; Kawamura, K.; Kim, Y. Dicarboxylic Acids, Oxocarboxylic Acids and A-Dicarbonyls in Fine Aerosols over Central Alaska: Implications for Sources and Atmospheric Processes. *Atmos. Res.* **2018**, 202, 128-139.
(39) Kawamura, K.; Sakaguchi, F. Molecular Distributions of Water Soluble Dicarboxylic Acids in Marine Aerosols over the Pacific Ocean Including Tropics. *J. Geophys. Res.* **1999**, 104, 3501-3509.
(40) Hoque, M.; Kawamura, K.; Seki, O.; Hoshi, N. Spatial Distributions of Dicarboxylic Acids, Ω-Oxoacids, Pyruvic Acid and A-Dicarbonyls in the Remote Marine Aerosols over the North Pacific. *Mar. Chem.* **2015**, 172, 1-11.
(41) Feltracco, M.; Barbaro, E.; Spolaor, A.; Vecchiato, M.; Callegaro, A.; Burgay, F.; Varde, M.; Maffezzoli, N.; Dallo, F.; Scoto, F.; Zangrando, R.; Barbante, C.; Gambaro, A. Year-Round Measurements of Size-Segregated Low Molecular Weight Organic Acids in Arctic Aerosol. *Sci. Total Environ.* **2021**, 763, 142954.
(42) Veres, P. R.; Neuman, J. A.; Bertram, T. H.; Assaf, E.; Wolfe, G. M.; Williamson, C. J.; Weinzierl, B.; Tilmes, S.; Thompson, C. R.; Thames, A. B.; Schroder, J. C.; Saiz-Lopez, A.; Rollins, A. W.; Roberts, J. M.; Price, D.; Peischl, J.; Nault, B. A.; Moller, K. H.; Miller, D. O.; Meinardi, S.; Li, Q. Y.; Lamarque, J. F.; Kupc, A.; Kjaergaard, H. G.; Kinnison, D.; Jimenez, J. L.; Jernigan, C. M.; Hornbrook, R. S.; Hills, A.; Dollner, M.; Day, D. A.; Cuevas, C. A.; Campuzano-Jost, P.; Burkholder, J.; Bui, T. P.; Brune, W. H.; Brown, S. S.; Brock, C. A.; Bourgeois, I.; Blake, D. R.; Apel, E. C.; Ryerson, T. B. Global Airborne Sampling Reveals a Previously Unobserved Dimethyl Sulfide Oxidation Mechanism in the Marine Atmosphere. *Proc. Natl. Acad. Sci. U.S.A.* **2020**,

117, 4505-4510.
(43) Walker, S. J.; Evans, M. J.; Jackson, A. V.; Steinbacher, M.; Zellweger, C.; McQuaid, J. B. Processes Controlling the Concentration of Hydroperoxides at Jungfraujoch Observatory, Switzerland. *Atmos. Chem. Phys.* **2006**, 6, 5525-5536.
(44) Kilgour, D. B.; Novak, G. A.; Sauer, J. S.; Moore, A. N.; Dinasquet, J.; Amiri, S.; Franklin, E. B.; Mayer, K.; Winter, M.; Morris, C. K.; Price, T.; Malfatti, F.; Crocker, D. R.; Lee, C.; Cappa, C. D.; Goldstein, A. H.; Prather, K. A.; Bertram, T. H. Marine Gas-Phase Sulfur Emissions During an Induced Phytoplankton Bloom. *Atmos. Chem. Phys.* **2022**, 22, 1601-1613.
(45) Lu, T.; Chen, F. Multiwfn: A Multifunctional Wavefunction Analyzer. *J. Comput. Chem.* **2012**, 33, 580-592.
